# A Novel Protocol for the Synthesis of 1,2,4-Oxadiazoles Active against Trypanosomatids and Drug-Resistant Leukemia Cell Lines

**DOI:** 10.3390/tropicalmed7120403

**Published:** 2022-11-28

**Authors:** Paulo Pitasse-Santos, Eduardo Salustiano, Raynná Bittencourt Pena, Otávio Augusto Chaves, Leonardo Marques da Fonseca, Kelli Monteiro da Costa, Carlos Antônio do Nascimento Santos, Jhenifer Santos Dos Reis, Marcos André Rodrigues da Costa Santos, Jose Osvaldo Previato, Lucia Mendonça Previato, Leonardo Freire-de-Lima, Nelilma Correia Romeiro, Lúcia Helena Pinto-da-Silva, Célio G. Freire-de-Lima, Débora Decotè-Ricardo, Marco Edilson Freire-de-Lima

**Affiliations:** 1 Instituto de Química, Universidade Federal Rural do Rio de Janeiro, Seropédica 23890-000, Rio de Janeiro, Brazil; 2 Instituto de Biofísica Carlos Chagas Filho, Universidade Federal do Rio de Janeiro, Rio de Janeiro 21491-170, Rio de Janeiro, Brazil; 3 Laboratório Integrado de Computação Científica (LICC), Universidade Federal do Rio de Janeiro—Centro Multidisciplinar UFRJ Macaé, Macaé 27930-560, Rio de Janeiro, Brazil; 4 Laboratório de Imunofarmacologia, Instituto Oswaldo Cruz (IOC), Fundação Oswaldo Cruz (Fiocruz), Rio de Janeiro 21040-900, Rio de Janeiro, Brazil; 5 Coimbra Chemistry Center, Departamento de Química, Institute of Molecular Sciences, Universidade de Coimbra, Rua Larga s/n, 3000 Coimbra, Portugal; 6 Instituto de Veterinária, Universidade Federal Rural do Rio de Janeiro, Seropédica 23890-000, Rio de Janeiro, Brazil

**Keywords:** chagas disease, *Trypanosoma cruzi*, *Leishmania amazonensis*, anticancer, chronic myeloid leukemia, molecular docking

## Abstract

Cancer and parasitic diseases, such as leishmaniasis and Chagas disease, share similarities that allow the co-development of new antiproliferative agents as a strategy to quickly track the discovery of new drugs. This strategy is especially interesting regarding tropical neglected diseases, for which chemotherapeutic alternatives are extremely outdated. We designed a series of (*E*)-3-aryl-5-(2-aryl-vinyl)-1,2,4-oxadiazoles based on the reported antiparasitic and anticancer activities of structurally related compounds. The synthesis of such compounds led to the development of a new, fast, and efficient strategy for the construction of a 1,2,4-oxadiazole ring on a silica-supported system under microwave irradiation. One hit compound (**23**) was identified during the in vitro evaluation against drug-sensitive and drug-resistant chronic myeloid leukemia cell lines (EC_50_ values ranging from 5.5 to 13.2 µM), *Trypanosoma cruzi* amastigotes (EC_50_ = 2.9 µM) and *Leishmania amazonensis* promastigotes (EC_50_ = 12.2 µM) and amastigotes (EC_50_ = 13.5 µM). In silico studies indicate a correlation between the in vitro activity and the interaction with tubulin at the colchicine binding site. Furthermore, ADMET in silico predictions indicate that the compounds possess a high druggability potential due to their physicochemical, pharmacokinetic, and toxicity profiles, and for hit 23, it was identified by multiple spectroscopic approaches that this compound binds with human serum albumin (HSA) via a spontaneous ground-state association with a moderate affinity driven by entropically and enthalpically energies into subdomain IIA (site I) without significantly perturbing the secondary content of the protein.

## 1. Introduction

Chagas disease and leishmaniasis are considered tropical neglected diseases by the World Health Organization (WHO) [1]. They are both parasitoses caused by flagellated protozoans from the *Trypanosomatidae* family, *Trypanosoma cruzi*, and *Leishmania* spp. The etiological agents have complex life cycles, infecting vertebrate and invertebrate hosts. Infected insects can also act as vectors for the parasites: kissing bugs (from subfamily *Triatominae*), which transmit *T. cruzi*, and sandflies (from subfamily *Phlebotominae*), which transmit *Leishmania* spp. In both cases, the most clinically relevant parasite form in the vertebrate host is the amastigote, which occurs intracellularly and is responsible for infection maintenance and chronicity. A critical common point between Chagas disease and leishmaniasis is the scarcity of therapeutic alternatives. The options that are available are ineffective and associated with severe side effects, and cases of the development of drug resistance have been reported [2,3].

Cancer and parasitic diseases, such as leishmaniasis and Chagas disease, share similarities that have been exploited in drug design and the development of new therapeutic strategies. The common features between cancer and parasitic illnesses include accelerated cell multiplication rates, multifactorial development of drug resistance, and the occurrence of mechanisms for evading the host immune system [4]. Such similarities have been driving drug repurposing in both directions [5,6,7,8]. Miltefosine, which is currently the only oral treatment approved for leishmaniasis [9], was originally used for the treatment of colorectal cancer and cutaneous metastases in breast cancer [10,11]. A different approach, aiming to quickly track the development of new drugs, would be designing new antiproliferative agents with both anticancer and antiparasitic activity [12,13,14]. In this work, we exploited this strategy using the (*E*)-3-phenyl-5-(2-aryl-vinyl)-1,2,4-oxadiazole motif as a scaffold.

The antiproliferative activity of compounds possessing the (*E*)-2-aryl-vinyl motif against cancer cell lines and protozoan species has been widely reported. For instance, cinnamamide derivative **1** induced 58.1% parasite death on *T. cruzi* epimastigotes at 50 μM (40.2% apoptosis and 17.9% necrosis) [15]. Additionally, phenylcinnamamide 2 was described as a potent antimitotic agent against sensitive and drug-resistant promyelocytic leukemias (IC_50_ = 3.1 μM, HL-60, IC_50_ = 11.6 μM, HL-60/VCR), acting through microtubule destabilization [16]. A chalcone heterocycle analog (3) presented activity against *Leishmania amazonensis* (IC_50_ = 0.6 μM) [17], and chalcone derivative **4** displayed a potent antitrypanosomal profile against *T. cruzi* amastigotes (IC_50_ = 5.6 μM) [18]. Multidrug resistance (MDR) in cancer cells and parasitic protozoa is often associated with the overexpression of proteins of the ATP binding cassette (ABC) transporter proteins [19,20]. A series of derivatives, analogous to goniothalamin (**5**), have been found to reverse MDR in cancer cells in cotreatment with doxorubicin [21].

**Figure 1:**
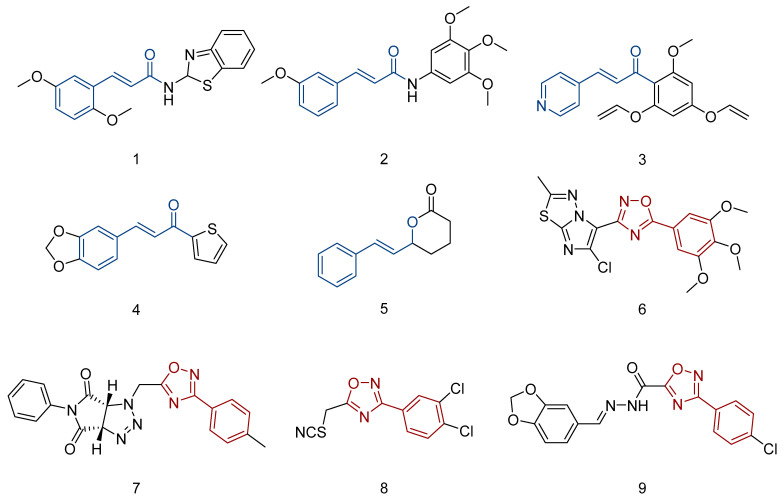
Chemical structures of compounds with described antitrypanosomal and antimitotic activity bearing the (*E*)-2-aryl-vinyl (in blue) or the 3-aryl-1,2,4-oxadiazole (in red) moieties.

1,2,4-Oxadiazoles have been explored in medicinal chemistry for the development of bioactive compounds [22,23]. They are useful tools for drug design due to their molecular rigidity, metabolic stability, and bioisoster substitutes for labile carbonyl groups such as esters, amides, and carbamates [24]. Active compounds containing the 1,2,4-oxadiazole core have also been described for their antiproliferative activity against cancer cell lines and parasites. The 1,2,4-oxadiazole-imidazothiazole derivative (**6**) presents in vitro anticancer activity toward diverse cancer cell lines (A375, IC_50_ = 1.22 μM, MCF-7, IC_50_ = 0.23 μM; ACHN, IC_50_ = 0.11 μM) [25]. The anti-protozoal activity of 1,2,4-oxadiazole derivative **7** was described against *Trypanosoma brucei rhodesiense* (IC_50_ = 21.6 μM), *T. cruzi* (IC_50_ = 100.2 μM) and *Leishmania donovani* (IC_50_ = 5.7 μM) [26]. Compound **8** was able to inhibit the in vitro growth of protozoan (*L. donovani*, IC_50_ = 2.3 μM; *T. brucei*, IC_50_ = 5.2 μM) and cancer cells (PC3, IC_50_ = 3.9 μM; Vero, IC_50_ = 70.0 μM) [27]. The *N*-acylhydrazone-1,2,4-oxadiazole conjugate 9 displayed antiprotozoal activity against *T. cruzi* (trypomastigotes, IC_50_ = 3.5 μM), and it can inhibit infectivity in vivo and reduce parasitemia in infected mice [28]. The chemical structures of compounds **1**–**9** are shown in Figure 1.

**Figure 2:**
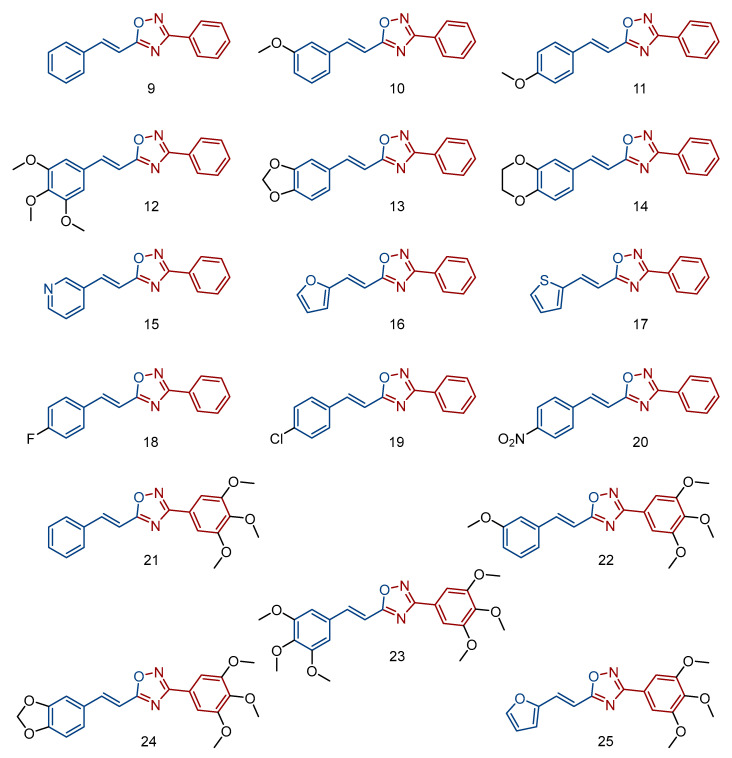
Chemical structures of (*E*)-3-aryl-5-(2-aryl-vinyl)-1,2,4-oxadiazoles (**9**–**25**) planned for this work.

Based on the reported activity panel, our research group proposed a series of 3-aryl-5-(2-aryl-(*E*)-vinyl)-1,2,4-oxadiazoles (**9**–**25**, Figure 2) with potential antiproliferative activity against neoplastic cell lines and trypanosomatids. The compounds were designed using a molecular hybridization approach. Although there are several protocols describing the synthesis of 1,2,4-oxadiazoles [29,30,31], we herein present a novel methodology using silica gel as a solid support under microwave irradiation to build the heterocycle group. We synthesized chemically diverse 1,2,4-oxadiazole derivatives bearing aromatic rings substituted with electron donors or withdrawing groups, as well as heteroaromatic ring systems. The compounds were assessed against sensitive and drug-resistant models of human chronic myeloid leukemia (CML), *T. cruzi* amastigotes, and *L. amazonensis* promastigotes and amastigotes for their antiproliferative activity. To better understand the biological activity profile, the binding interaction with tubulin was investigated in silico. Additionally, the absorption, distribution, metabolization, excretion, and toxicity (ADMET) profiles were predicted, and the experimental binding interaction with human serum albumin (HSA) was studied to complement the proposed pharmacokinetic predictions.

## 2. Materials and Methods

### 2.1. General Procedures

All starting materials and reagents for chemical synthesis, drug standards for cell experiments, commercially available HSA (lyophilized powder, fatty acid-free, globulin free with purity higher than 99%, code A3782-1G), phosphate buffered saline (PBS, pH = 7.4), warfarin, ibuprofen, and digitoxin were purchased from Sigma-Aldrich (St. Louis, MO, USA). Solvents were purchased from Neon Comercial (Suzano, SP, Brazil). Thin layer chromatography (TLC) was performed on silica gel G60 F254 plates, and chromatograms were visualized by using UV light (λ = 254 and 365 nm). Column chromatography was performed using silica gel 60 (40−63 mm, 230−400 mesh). Melting points were recorded manually. A Bruker (Billerica, MA, USA) Vertex 70 spectrophotometer was employed to record IR spectra. Chemical shifts are reported in parts per million (δ in ppm) and coupling constants in hertz (*J* in Hz). The nuclear magnetic resonance (NMR) spectra were recorded either on Bruker 500 MHz or Bruker 400 MHz using CDCl_3_ as solvent and internal standard at 7.26 ppm for ^1^H NMR and 77.16 ppm for ^13^C NMR (Figures S1–S34). High-resolution mass spectra were recorded on a Waters Micromass Q-TOF. Fetal bovine serum (FBS) was purchased from Cultilab (São Paulo, SP, Brazil) and inactivated at 56 °C for 1 h prior to use. All other cell culture media, supplements, and reagents were purchased from Gibco Thermo Fisher (Waltham, MA, USA). Compounds were dissolved in 100% dimethylsulfoxide (DMSO) at 30 mM and then diluted to the final concentrations with the suitable culture media for each experiment. The maximum concentration of DMSO in the experiments was 0.3% *v*/*v*, which did not interfere with cell development. Absorbance was recorded using a BioRad (Hercules, CA, USA) iMark microplate reader. Six- to eight-week-old BALB/c mice were obtained from Veterinary Institute/UFRRJ, Brazil. The animals were housed in polypropylene boxes with free access to water and standard feed at a controlled temperature (23–25 °C) during the experiments. The mice were used to obtain splenocytes.

### 2.2. Synthesis of Benzamidoximes ***27a**–**b***

A mixture of the properly substituted benzonitrile (**26a**–**b**) (9.7 mmol) and 5 mg (34 nmol) 8-hydroxyquinoline were diluted in 8.0 mL ethanol. Two solutions containing 1.4 g (20.1 mmol) hydroxylamine hydrochloride and 2.1 g (15.2 mmol) potassium carbonate in 4 mL water were simultaneously added dropwise for 10 min under stirring at room temperature. Next, the reaction was heated until reflux for 1 h. After cooling to room temperature, the solvent was removed, and the resulting solid was suspended in 150 mL of ethyl acetate and washed with 4 × 250 mL of brine. The organic phase was then dried over 50 g sodium sulfate, and the solvent was removed to afford the pure products. The chemical characterization data for 27a and 27b have been previously reported [32,33].

### 2.3. Synthesis of (E)-3-aryl-acryloyl chlorides ***29a**–**l***

The suitable (*E*)-3-aryl-acrylic acid (**28a**–**l**) (1.37 mmol) was diluted in oxalyl chloride (2.0 mL), and the reaction was stirred at room temperature in a sealed vessel. The reaction was monitored by TLC through the visualization of the corresponding methyl ester produced after the addition of an aliquot of the reaction mixture to 200 µL methanol. After completion of the reaction, the solvent was removed under reduced pressure in an anhydrous system. The products were used without further purification or characterization due to their chemical instability to humidity.

### 2.4. Synthesis of (E)-3-phenyl-5-(2-aryl-vinyl)-1,2,4-oxadiazoles *(**9**–**25**)*

The suitable benzamidoxime (**27a**–**b**) (1.14 mmol) and 350 mg (2.53 mmol) of dry potassium carbonate were added to a sealed vessel containing 3.0 mL of anhydrous dichloromethane under a dry N_2_ atmosphere. Then, the suitable 3-aryl-acryloyl chlorides (**29a**–**l**) were diluted in 3.0 mL anhydrous dichloromethane and added dropwise under stirring at room temperature. After complete consumption of the reagents, 1 g silica gel (60–120 mesh) was added, and the solvent was removed under low pressure. The solid-supported reaction was then conducted under microwave irradiation (75 W, 100–105 °C) over 5–45 min (monitored by TLC). The pure products were obtained after column chromatography in silica gel (60–120 mesh) eluted with hexanes/ethyl acetate (9:1).

#### 2.4.1. (*E*)-3-phenyl-5-[2-phenylvinyl]-1,2,4-oxadiazole **9**

White solid, weight = 192.5 mg (0.78 mmol, yield 68%); m.p. 85–87 °C; ^1^H NMR (500 MHz, CDCl_3_) δ ppm: 7.11 (d, *J* 16 Hz, 1H), 7.46–7.48 (m, 3H), 7.53–7.56 (m, 3H), 7.64–7.66 (m, 3H), 7.92 (d, *J* 16 Hz, 1H), 8.15–8.17 (m, 2H); ^13^C NMR: (125 MHz, CDCl_3_) δ ppm: 110.25, 126.95, 127.47, 127.96, 128.88, 129.10, 130.56, 131.19, 134.43, 142.74, 168.74, 175.26; IR (cm^−1^): 960.52, 1359.77, 1440.78, 1539.15, 1577.72, 1641.37, 3028.15, 3062.86; HRMS (ESI^+^) for C_16_H_13_N_2_O^+^ [M+H]^+^, calcd. 249.1022 found 249.1028. Product characterization was consistent with the available data [34].

#### 2.4.2. (*E*)-3-phenyl-5-[2-(3-methoxyphenyl)vinyl]-1,2,4-oxadiazole **10**

White solid, weight = 237.9 mg (0.86 mmol, yield 75%); m.p. 88–90 °C; ^1^H NMR (500 MHz, CDCl_3_) δ ppm: 3.89 (s, 3H), 7.00 (dd, *J*_1_ 8 Hz, *J*_2_ 2 Hz, 1H), 7.09 (d, *J* 15 Hz, 1H), 7.15 (t, *J* 2 Hz, 1H), 7.24 (dd, *J*_1_ 8 Hz, *J*_2_ 2 Hz), 7.38 (t, *J* 8 Hz, 1H), 7.51–7.55 (m, 3H), 7.89 (d, *J* 15 Hz, 1H), 8.15 (dd, *J*_1_ 8 Hz, *J*_2_ 2 Hz, 2H); ^13^C NMR: (125 MHz, CDCl_3_) δ ppm: 55.37, 110.53, 112.83, 116.41, 120.63, 126.92, 127.46, 128.88, 130.10, 131.19, 135.77, 142.65, 160.03, 168.74, 175.19; IR (cm^−1^): 966.3, 1049.24, 1265.26, 1363.63, 1442.71, 1556.51, 1606.65, 1637.51, 2835.27, 2956.78, 3010.79, 3084.08; HRMS (ESI^+^) for C_17_H_15_N_2_O_2_^+^ [M+H]^+^, calcd. 279.1128 found 279.1133.

#### 2.4.3. (*E*)-3-phenyl-5-[2-(4-methoxyphenyl)vinyl]-1,2,4-oxadiazole **11**

White solid, weight = 190.4 mg (0.68, yield: 65%); m.p. 132–135 °C; ^1^H NMR (500 MHz, CDCl_3_) δ ppm: 3.89 (s, 3H), 6.96 (d, *J* 15 Hz, 1H), 6.98 (d, *J* 8 Hz), 7.50–7.53 (m, 3H), 7.59 (d, *J* 8 Hz, 2H), 7.87 (d, *J* 15 Hz, 1H), 8.15 (dd, *J*_1_ 8 Hz, *J*_2_ 2 Hz, 2H); ^13^C NMR: (125 MHz, CDCl_3_) δ ppm: 55.45, 107.76, 114.54, 127.07, 127.20, 127.44, 128.85, 129.64, 131.10, 142.37, 161.60, 168.63, 175.63; IR (cm^−1^): 966.22, 1026.01, 1245.87, 1361.59, 1441.62, 1537.09, 1599.77, 1639.30, 2840.82, 2972.92, 3005.71, 3069.35; HRMS (ESI^+^) for C_17_H_15_N_2_O_2_^+^ [M+H]^+^, calcd. 279.1128 found 279.1133.

#### 2.4.4. (*E*)-3-phenyl-5-[2-(3,4,5-trimethoxyphenyl)vinyl]-1,2,4-oxadiazole **12**

Pale brown solid, weight = 270 mg (0.80 mmol, yield 70%); m.p. 132–135 °C; ^1^H NMR (500 MHz, CDCl_3_) δ ppm: 3.93 (s, 3H), 3.95 (s, 6H), 6.87 (s, 1H), 7.01 (d, *J* 16 Hz, 1H), 7.51–7.55 (m, 3H), 7.83 (d, *J* 16 Hz, 1H), 8.15 (dd, *J*_1_ 8 Hz, *J*_2_ 2 Hz, 2H); ^13^C NMR: (125 MHz, CDCl_3_) δ ppm: 56.21, 61.06, 105.04, 109.49, 126.93, 127.43, 128.90, 129.93, 131.21, 140.30, 142,64, 153.58, 168.70, 175.22; IR (cm^−1^): 995.15, 1128.23, 1240.08, 1336.51, 1415.51, 1548.66, 1581.44, 1625.80, 2833.10, 2991.25, 3066.46; ESI-HRMS (m/z): HRMS (ESI^+^) for C_19_H_19_N_2_O_4_^+^ [M+H]^+^, calcd. 339.1339 found 339.1358.

#### 2.4.5. (*E*)-3-phenyl-5-[2-(3,4-methylenedioxy-phenyl)vinyl]-1,2,4-oxadiazole **13**

White solid, weight = 213.3 mg (0.73 mmol, yield 64%); m.p. 157–159 °C; ^1^H NMR (500 MHz, CDCl_3_) δ ppm: 6.06 (s, 2H), 6.89 (d, *J* 8 Hz, 1H), 6.91 (d, *J* 15 Hz, 1H), 7.12 (dd, *J*_1_ 8 Hz, *J*_2_ 2 Hz, 1H), 7.15 (d, *J* 2 Hz, 1H), 7.51–7.54 (m, 3H), 7.82 (d, *J* 15 Hz, 1H), 8.13–8.15 (m, 2H); ^13^C NMR: (125 MHz, CDCl_3_) δ ppm: 101.70, 106.22, 108.20, 108.74, 124.53, 127.01, 127.44, 128.85, 128.90, 131.13, 142.37, 148.56, 149.88, 168.66, 175.44; IR (cm^−1^): 967.19, 1032.76, 1245.87, 1349.05, 1441.62, 1553.48, 1596.87, 1635.45, 2850.46, 2950.74, 3021.14, 3066.46; HRMS (ESI^+^) for C_17_H_13_N_2_O_3_^+^ [M+H]^+^, calcd. 293.0921 found 293.0926.

#### 2.4.6. (*E*)-3-phenyl-5-[2-(3,4-ethylenedioxy-phenyl)vinyl]-1,2,4-oxadiazole **14**

White solid, weight = 244.4 mg (0.80 mmol, yield): 70%; m.p. 143–145 °C; ^1^H NMR (500 MHz, CDCl_3_) δ ppm: 4.32 (m, 4H), 6.92 (d, *J* 8 Hz, 1H), 6.93 (d, *J* 15 Hz, 1H), 7.15 (dd, *J*_1_ 8 Hz, *J*_2_ 2 Hz, 1H), 7.17 (d, *J* 2 Hz, 1H), 7.51–7.54 (m, 3H), 7.78 (d, *J* 15 Hz, 1H), 8.13–8.15 (m, 2H); ^13^C NMR: (125 MHz, CDCl_3_) δ ppm: 64.24, 64.60, 108.36, 116.55, 117.90, 121.98, 127.03, 127.44, 128.13, 128.85, 131.11, 142.29, 143.86, 145.92, 168.64, 175.50; IR (cm^−1^): 980.69, 1057.83, 1244.91, 1284.44, 1360.62, 1443.55, 1538.05, 1604.59, 1644.12, 2876.49, 2978.71, 3040.52, 3058.75; HRMS (ESI^+^) for C_18_H_15_N_2_O_3_^+^ [M+H]^+^, calcd. 307.1077 found 307.1083.

#### 2.4.7. (*E*)-3-phenyl-5-[2-pyridyn-3-yl-vinyl]-1,2,4-oxadiazole **15**

Pale brown solid, weight = 193.2 (0.78 mmol, yield 68%); m.p. 107–110 °C; ^1^H NMR (500 MHz, CDCl_3_) δ ppm: 7.17 (d, *J* 16 Hz, 1H), 7.42 (dd, *J*_1_ 8 Hz, *J*_2_ 4 Hz, 1H), 7.51–7.57 (m, 3H), 7.91 (d, *J* 16 Hz, 1H), 7.96 (dt, *J*_1_ 8 Hz, *J*_2_ 2 Hz, 1H), 8.15 (dd, *J*_1_ 8 Hz, *J*_2_ 4 Hz, 2H), 8.67 (dd, *J*_1_ 8 Hz, *J*_2_ 2 Hz, 1H), 8.87 (d, *J* 2 Hz, 1H); ^13^C NMR: (125 MHz, CDCl_3_) δ ppm: 112.34, 123.92, 126.73, 127.47, 128.93, 130.24, 131.32, 133.95, 138.95, 149.69, 151.23, 168.86, 174.56; IR (cm^−1^): 972.01, 1024.08, 1116.65, 1292.16, 1355.80, 1417.51, 1444.52, 1542.87, 1643.16, 3033.67, 3056.82; HRMS (ESI^+^) for C_15_H_12_N_3_O^+^ [M+H]^+^, calcd. 250.0975 found 250.0964.

#### 2.4.8. (*E*)-3-phenyl-5-[2-fur-2-yl-vinyl]-1,2,4-oxadiazole **16**

Pale brown solid, weight = 165.7 (0.70 mmol, yield 61%); m.p. 83–85 °C; ^1^H NMR (500 MHz, CDCl_3_) δ ppm: 6.55 (m, 1H), 6.72 (d, *J* 5 Hz, 1H), 6.97 (d, *J* 15 Hz, 1H), 7.50–7.57 (m, 4H), 7.78 (d, *J* 15 Hz, 1H), 8.13–8.15 (dd, *J*_1_ 8 Hz, *J*_2_ 2 Hz, 2H); ^13^C NMR: (125 MHz, CDCl_3_) δ ppm: 107.85, 112.59, 115.22, 126.98, 127.44, 128.80, 128.85, 131.14, 145.12, 150.81, 168.73, 175.28; IR (cm^−1^): 958.59, 1261.41, 1290.34, 1359.77, 1442.71, 1527.58, 1583.51, 1641.37, 3066.72, 3124.59; HRMS (ESI^+^) for C_14_H_11_N_2_O_2_^+^ [M+H]^+^, calcd. 239.0815 found 239.0820.

#### 2.4.9. (*E*)-3-phenyl-5-[2-thiophen-2-yl-vinyl]-1,2,4-oxadiazole **17**

Pale yellow solid, weight = 165.3 mg (0.65 mmol, yield 57%); m.p. 93–95 °C; ^1^H NMR (500 MHz, CDCl_3_) δ ppm: 6.89 (d, *J* 16 Hz, 1H), 7.13 (t, *J* 5 Hz, 1H), 7.37 (d, *J* 5 Hz, 1H), 7.47 (d, *J* 5 Hz, 1H), 7.51–7.56 (m, 3H), 8.01 (d, *J* 16 Hz, 1H), 8.13–8.15 (m, 2H); ^13^C NMR: (125 MHz, CDCl_3_) δ ppm: 108.98, 126.94, 127.44, 128.35, 128.87, 131.08, 131.17, 135.15, 139.72, 168.70, 175.03; IR (cm^−1^): 956.66, 1359.77, 1444.64, 1502.50, 1552.65, 1635.59, 3059.01, 3097.58; HRMS (ESI^+^) for C_14_H_11_N_2_OS^+^ [M+H]^+^, calcd. 255.0587 found 255.0592.

#### 2.4.10. (*E*)-3-phenyl-5-[2-(4-fluorophenyl)vinyl]-1,2,4-oxadiazole **18**

White solid, weight = 167.0 mg (0.63 mmol, yield 55%); m.p. 105–107 °C; ^1^H NMR (500 MHz, CDCl_3_) δ ppm: 7.02 (d, *J* 16 Hz, 1H), 7.16 (dd, *J*_1_ 5 Hz, *J*_2_ 5 Hz, 2H); 7.47–7.57 (m, 3H), 7.63 (dd, *J*_1_ 8 Hz, *J*_2_ 5 Hz, 2H); 7.88 (d, *J* 16 Hz, 1H), 8.15 (dd, *J*_1_ 8 Hz, *J*_2_ 2 Hz, 2H); ^13^C NMR: (125 MHz, CDCl_3_) δ ppm: 107.75, 116.29 (d, ^2^*J*_C–F_ 22 Hz), 126.87, 127.45, 128.89, 129.85 (d, ^3^*J*_C–F_ 9 Hz), 130.69 (d, ^4^*J*_C–F_ 3 Hz), 131.22, 141.39, 164.03 (d, ^1^*J*_C–F_ 250 Hz), 168.72, 175.10; IR (cm^−1^): 974.02, 1224.76, 1359.77, 1442.71, 1544.93, 1595.08, 1643.30, 3049.36; HRMS (ESI^+^) for C_16_H_12_FN_2_O^+^ [M+H]^+^, calcd. 267.0928 found 267.0934.

#### 2.4.11. (*E*)-3-phenyl-5-[2-(4-chlorophenyl)vinyl]-1,2,4-oxadiazole **19**

White solid, weight = 161.2 mg (0.63 mmol, yield 50%); m.p. 151–153 °C; ^1^H NMR (500 MHz, CDCl_3_) δ ppm: 7.07 (d, *J* 16 Hz, 1H), 7.44 (d, *J* 8 Hz, 2H); 7.52–7.58 (m, 5H), 7.87 (d, *J* 16 Hz, 1H), 8.14–8.15 (m, 2H); ^13^C NMR: (125 MHz, CDCl_3_) δ ppm: 110.78, 126.85, 127.45, 128.90, 129.08, 129.40, 131.24, 132.90, 136.50, 141.25, 168.79, 174.97; IR (cm^−1^): 815.86, 975.95, 1361.70, 1444.64, 1543.01, 1571.94, 1645.23, 3037.79, 3060.94; HRMS (ESI^+^) for C_16_H_12_ClN_2_O^+^ [M+H]^+^, calcd. 283.0633 found 283.0638. Product characterization was consistent with the available data [34].

#### 2.4.12. (*E*)-3-phenyl-5-[2-(4-nitrophenyl)vinyl]-1,2,4-oxadiazole **20**

Pale yellow solid, weight = 264.1 mg (0.9 mmol, yield 79%); m.p. 205–208 °C; ^1^H NMR (500 MHz, CDCl_3_) δ ppm: 7.24 (d, *J* 16 Hz, 1H), 7.52–7.58 (m, 3H), 7.80 (d, *J* 8 Hz, 2H), 7.95 (d, *J* 16 Hz, 1H), 8.14–8.15 (m, 2H), 8.33 (d, *J* 8 Hz, 2H); ^13^C NMR: (125 MHz, CDCl_3_) δ ppm: 114.34, 124.39, 126.59, 127.47, 128.52, 128.96, 131.42, 139.69, 140.41, 148.59, 168.99, 174.25; IR (cm^−1^): 975.87, 1361.70, 1512.98, 1445.48, 1548.66, 1595.91, 1645.09, 3034.64, 3108.89; HRMS (ESI^+^) for C_16_H_12_N_3_O_3_^+^ [M+H]^+^, calcd. 294.0873 found 294.0879.

#### 2.4.13. (*E*)-3-(3,4,5-trimethoxyphenyl)-5-[2-phenylvinyl]-1,2,4-oxadiazole **21**

White solid, weight = 281.3 (0.86 mmol, yield 73%); m.p. 138–140 °C; ^1^H NMR (400 MHz, CDCl_3_) δ ppm: 3.95 (s, 3H), 3.99 (s, 6H, 7.10 (d, *J* 16 Hz, 1H), 7.52–7.58 (m, 3H), 7.80 (d, *J* 8 Hz, 2H), 7.40 (s, 2H), 7.46–7.48 (m, 3H), 7.63–7.66 (m, 2H), 7.92 (d, *J* 16 Hz, 1H); ^13^C NMR: (500 MHz, CDCl_3_) δ ppm: 56.31, 61.00, 104.52, 110.15, 122.14, 127.95, 129.12, 130.62, 134.39, 140.50, 142.87, 153.57, 168.57, 175.21; IR (cm^−1^): 995.15, 1129.19, 1229.48, 1363.51, 1413.66, 1557.34, 1595.95, 1636.41, 2832.14, 2962.32, 3037.79, 3060.94; HRMS (ESI^+^) for C_19_H_19_N_2_O_4_^+^ [M+H]^+^, calcd. 339.1339 found 339.1345.

#### 2.4.14. (*E*)-3-(3,4,5-trimethoxyphenyl)-5-[2-(3-methoxyphenyl)-vinyl]-1,2,4-oxadiazole **22**

White solid, weight = 252.0 mg (0.68 mmol, yield 60%); m.p. 117–119 °C; ^1^H NMR (500 MHz, CDCl_3_) δ ppm: 3.88 (s, 3H), 3.94 (s, 3H), 3.99 (s, 6H), 7.00 (dd, *J*_1_ 5 Hz, *J*_2_ 2 Hz, 1H), 7.08 (d, *J* 16 Hz, 1H), 7.15 (sl, 1H), 7.22 (dl, *J* 5 Hz, 1H), 7.37 (d, *J* 5 Hz, 1H), 7.40 (s, 2H), 7.88 (d, *J* 16 Hz, 1H); ^13^C NMR: (125 MHz, CDCl_3_) δ ppm: 55.35, 56.30, 60.98, 104.51, 110.42, 112.85, 116.41, 120.62, 122.12, 130.11, 135.72, 140.49, 142.76, 153.56, 160.03, 168.55, 175.12; IR (cm^−1^): 974.02, 1128.32, 1230.55, 1361.70, 1417.64, 1554.58, 1602.80, 1641.37, 2831.41, 3033.93, 3062.86; HRMS (ESI^+^) for C_20_H_21_N_2_O_5_^+^ [M+H]^+^, calcd. 369.1445 found 369.1451.

#### 2.4.15. (*E*)-3-(3,4,5-trimethoxyphenyl)-5-[2-(3,4,5-trimethoxyphenyl)-vinyl]-1,2,4-oxadiazole **23**

Off-white solid, weight = 371.2 mg (0.86 mmol, yield 76%); m.p. 118–120 °C; ^1^H NMR (500 MHz, CDCl_3_) δ ppm: 3.93–3.94 (m, 12H), 3.98 (s, 6H), 6.86 (s, 2 Hz, 1H), 7.00 (d, *J* 16 Hz, 1H), 7.39 (s, 2H), 7.83 (d, *J* 16 Hz, 1H); ^13^C NMR: (125 MHz, CDCl_3_) δ ppm: 56.21, 56.30, 61.00, 61.06, 104.50, 105.08, 109.41, 122.12, 129.89, 140.40, 140.51, 142.76, 153.57, 160.03, 168.54, 175.16; IR (cm^−1^): 962.37, 995.15, 1120.51, 1230.44, 1336.51, 1415.59, 1485.02, 1583.37, 1647.02, 2835.03, 2937.24, 3000.89; HRMS (ESI^+^) for C_22_H_25_N_2_O_7_^+^ [M+H]^+^, calcd. 429.1656 found 429.1608.

#### 2.4.16. (*E*)-3-(3,4,5-trimethoxyphenyl)-5-[2-(3,4-methylenedioxy-phenyl)vinyl]-1,2,4-oxadiazole **24**

White solid, weight = 261.5 mg (0.68 mmol, yield 60%); m.p. 177–180 °C; ^1^H NMR (500 MHz, CDCl_3_) δ ppm: 3.93 (s, 3H), 3.95 (s, 6H), 6.86 (s, 2H), 7.01 (d, *J* 15 Hz, 1H), 7.51–7.55 (m, 3H), 7.83 (d, *J* 15 Hz, 1H), 8.14–8.16 (m, 2H); ^13^C NMR: (125 MHz, CDCl_3_) δ ppm: 59.30, 60.99, 101.73, 104.50, 106.20, 108.10, 108.75, 122.22, 124.56, 128.87, 142,51, 148.58, 149.52, 153.55, 168.49, 175.40; IR (cm^−1^): 996.12, 1032.76, 1130.15, 1245.87, 1230.44, 1354.84, 1414.62, 1539.98, 1597.84, 1647.02, 2836.96, 2983.53, 3009.57, 3103.10; HRMS (ESI^+^) for C_20_H_19_N_2_O_6_^+^ [M+H]^+^, calcd. 383.1238 found 383.1243.

#### 2.4.17. (*E*)-3-(3,4,5-trimethoxyphenyl)-5-[2-(fur-2-yl)vinyl]-1,2,4-oxadiazole **25**

Pale brown solid, weight = 228.3 (0.70 mmol, yield 62%); m.p. 144–146 °C; ^1^H NMR (500 MHz, CDCl_3_) δ ppm: 3.93 (s, 3H), 3.97 (s, 6H), 6.53–6.54 (m, 1H), 6.72 (d, *J* 5 Hz, 1H), 6.95 (d, *J* 16 Hz, 1H), 7.15 (s, 1H), 7.38 (s, 2H), 7.56 (s, 1H), 7.64 (d, *J* 16 Hz, 1H, =C–H); ^13^C NMR: (125 MHz, CDCl_3_) δ ppm: 56.04, 60.73, 104.25, 107.51, 112.35, 115.02, 121.94, 128.64, 140.20, 144.93, 150.53, 153.29, 168.30, 174.97; IR (cm^−1^): 972.01, 999.01, 1126.30, 1178.37, 1228.51, 1359.66, 1413.66, 1456.09, 1486.94, 1544.80, 1567.94, 1594.95, 1637.37, 2835.03, 2937.24; 2966.27, 3008.60, 3072.25, 3159.03; HRMS (ESI^+^) for C_17_H_17_N_2_O_5_^+^ [M+H]^+^, calcd. 329.1132 found 329.1107.

### 2.5. Cultivation and Development of Mammalian Cell Lineages

The CML cell lines K562, Lucena-1 (K562/VCR), and FEPS (K562/DNR) were cultured in RPMI-1640 medium supplemented with 25 mM HEPES and 2 g L^−1^ sodium bicarbonate adjusted to pH 7.4 with NaOH, 100 U penicillin and 100 g L^−1^ streptomycin. All media were supplemented with 10% FBS. Dr. Vivian M. Rumjanek (Instituto de Bioquímica Médica, UFRJ, Brazil) kindly donated Lucena-1 and FEPS cells. Briefly, K562 cells were exposed to increasing concentrations of the chemotherapeutic drugs vincristine sulfate (VCR) and daunorubicin hydrochloride (DNR) (both from Sigma-Aldrich), as described previously [35,36]. Lucena-1 (K562/VCR) and FEPS (K562/DNR) cells were cultured in the presence of either 60 nM VCR or 500 nM DNR to maintain the MDR phenotypes. For subcultures, cells were harvested every three days followed by washing with cold FBS, and a final concentration of 2 × 10^4^ cells mL^−1^ was maintained at 37 °C in 5% CO_2_.

LLC-MK2 kidney fibroblasts (ATCC) and RAW 264.7 macrophages (ATCC) were cultivated in culture flasks in complete DMEM with 5% FBS at 37 °C in 5% CO_2_. The LLC-MK2 cell monolayer was detached by treatment with trypsin-EDTA 0.05% for 5 min at 37 °C. The RAW cell monolayer was detached mechanically. In both cases, the cells were centrifuged at 480× *g* for 6 min and resuspended in complete DMEM with 5% FBS, and the cell density was determined by counting with trypan-blue dye exclusion on Neubauer chambers before use.

### 2.6. Cultivation of T. cruzi Trypomastigotes

To a monolayer of 1.0 × 10^7^ LLC-MK2 cells in a 150 cm^2^ culture flask were added 5.0 × 10^7^ trypomastigotes of *T. cruzi* expressing the β-galactose gene (Tulahuen C2C4 LacZ) in complete DMEM with 2.5% FBS. The infection was allowed to occur for 24 h, the supernatant was removed, and the cell monolayer was washed with PBS. Complete DMEM with 2.5% FBS was renewed every 2 days up to the sixth day after infection. Trypomastigote forms were collected daily from the supernatant between days 7 and 12 after infection, enriched by differential centrifugation (1500× *g* for 10 min, 5580× *g* for 20 min), and resuspended in complete DMEM with 5% FBS. Parasite density was determined by counting the mobile trypomastigotes on Neubauer chambers before use.

### 2.7. Cultivation of L. amazonensis Promastigotes

Promastigotes of *L. amazonensis* (MHOM/BR/75/Josefa) were cultured in Schneider’s insect medium (SIM) with 10% FBS. Parasites were harvested at the stationary growth phase for assessment against promastigotes and 24 h after cultures reached the stationary growth phase for infection assays. Parasite density was determined before use by counting the mobile promastigotes on Neubauer chambers after centrifugation at 3000× *g*.

### 2.8. Assessment of Cytotoxicity to CML Cell Lines

Cell viability was determined by the 3-(4,5-dimethylthiazol-2-yl)-2,5-diphenyltetrazolium (MTT) colorimetric assay. CML cells (2 × 10^4^ cells·mL^−1^) were incubated for 72 h at 37 °C, followed by treatment with compounds at a range of concentrations (100–1 μM). Negative controls were prepared with 0.5% DMSO. An MTT salt solution was added to each well to a final concentration of 0.5 g·L^−1^, and plates were incubated at 37 °C in 5% CO_2_ for 3 h. After centrifugation, 200 μL DMSO was added to dissolve the dark-blue formazan crystals formed by MTT reduction. Absorbance was measured on a microplate reader at 570 nm, which is directly proportional to formazan contents and indicative of living cells [37].

### 2.9. Anti-T. cruzi Amastigote Assessment

LLC-MK2 cells were seeded in a 96-well microplate (1.0 × 10^4^ cells per well) in complete DMEM with 5% FBS and incubated for 3 h at 37 °C in 5% CO_2_ for adhesion. The medium was renewed, and trypomastigote forms of *T. cruzi* (Tulahuen C2C4 LacZ strain, 2.0 × 10^5^ parasites per well) were added. After incubation for 24 h at 37 °C in 5% CO_2_, the cell monolayer was washed with PBS to remove noninternalized parasites. DMEM without phenol red and 5% FBS was added, and infected cells were treated with compounds at a range of concentrations in triplicate (50 μM or 100–1 μM). Untreated infected cells and noninfected LLC-MK2 cells were used as negative and positive controls, respectively. Vehicle control containing DMEM without phenol red and 0.3% DMSO was maintained for comparison. After incubation for 120 h at 37 °C in 5% CO_2_, a solution containing 0.5 M CPRG and 0.8% IGEPAL CA-630 in PBS (30 µL per well), followed by further incubation for 2 h at 37 °C in 5% CO_2_. Absorbance was measured using a microplate reader at 570 nm [38].

### 2.10. Cytotoxicity Assessment of LLC-MK2 Host Cells

LLC-MK2 cells were seeded in a 96-well microplate (1.0 × 10^4^ cells per well) in complete DMEM with 5% FBS (120 µL per well). After incubation for 24 h at 37 °C in 5% CO_2_, the medium was renewed, and compounds were added at a range of concentrations in triplicate (50 μM or 100–1 μM). Untreated cells and cells treated with DMEM and 10% DMSO were used as negative and positive controls, respectively. Vehicle control containing DMEM and 0.3% DMSO was maintained for comparison. After incubation for 120 h at 37 °C in 5% CO_2_, the medium was renewed, and MTT salt solution in PBS was added (3.0 mM, 20 µL per well). After incubation for 2 h at 37 °C in 5% CO_2_, the supernatant was removed, and MTT formazan was dissolved in DMSO (150 µL per well). Absorbance was measured using a microplate reader at 570 nm [37].

### 2.11. Anti-L. amazonensis Promastigote Assessment

*L. amazonensis* promastigotes were seeded into a 96-well microplate (2.0 × 10^5^ parasites per well) in SIM with 10% FBS. The compounds were added at a range of concentrations in triplicate (100–1 μM). Complete SIM media and amphotericin B (AmB) at 10 µM were used as negative and positive controls, respectively. A vehicle control containing SIM with 10% SFB media and 0.3% DMSO was maintained for comparison. The final volume of the experiment was 100 µL. After incubation for 48 h at 27 °C, MTT salt solution in PBS was added (3.0 mM, 20 µL per well). After incubation for 2 h at 27 °C, the MTT formazan was dissolved by adding 120 µL of solubilizing buffer (50% isopropanol, 40% acetate buffer pH 5.4, 10% Triton-X). Absorbance was measured using a microplate reader at 570 nm [39,40].

### 2.12. Anti-L. amazonensis Amastigote Assessment

RAW 264.7 cells were seeded in a 96-well microplate (2.0 × 10^4^ cells per well) in complete DMEM with 5% FBS and incubated for 3 h at 37 °C in 5% CO_2_ for cell adhesion. The medium was renewed, and *L. amazonensis* promastigotes (3.0 × 10^5^ parasites per well) were added. After 24 h of incubation at 37 °C in 5% CO_2_, the cell monolayer was washed with PBS to remove noninternalized parasites, the medium was renewed, and compounds were added at a range of concentrations in triplicate (100–1 μM). Untreated infected cells and noninfected RAW 264.7 cells were used as negative and positive controls, respectively. Vehicle control containing DMEM and 0.3% DMSO was maintained for comparison. After incubation for 48 h at 37 °C in 5% CO_2_, the medium was removed, and a solution containing 0.02% SDS in PBS (20 µL per well) was added. Macrophage lysis and amastigote release were accompanied under an inverted microscope for 10–15 min. SIM with 12.5% SFB (80 µL per well) was added, parasites were allowed to differentiate into promastigotes for 48 h at 27 °C, and an MTT salt solution in PBS was added (3.0 mM, 20 µL per well). After incubation for 2 h at 27 °C, the MTT formazan was dissolved by adding 120 µL of solubilizing buffer (50% isopropanol, 40% acetate buffer pH 5.4, 10% Triton X). Absorbance was measured using a microplate reader at 570 nm [39,40].

### 2.13. Cytotoxicity Assessment to RAW Cells

RAW 264.7 cells were seeded in a 96-well microplate (2.0 × 10^4^ cells per well) in complete DMEM with 5% FBS (120 µL per well). After incubation for 24 h at 37 °C in 5% CO_2_, the medium was renewed, and compounds were added at a range of concentrations in triplicate (100–1 μM). Untreated cells and cells treated with DMEM and 10% DMSO were used as negative and positive controls, respectively. Vehicle control containing DMEM and 0.3% DMSO was maintained for comparison. After incubation for 48 h at 37 °C in 5% CO_2_, the medium was renewed, and an MTT solution in PBS was added (3.0 mM, 20 µL per well). After incubation for 2 h at 37 °C in 5% CO_2_, the supernatant was removed, and MTT formazan was dissolved in DMSO (150 µL per well). Absorbance was measured using a microplate reader at 570 nm [37].

### 2.14. Cytotoxicity Assessment of BALB/c Mouse Total Splenocytes

BALB/c mouse total splenocytes were harvested from male mice between 6–8 weeks old. After euthanasia, the spleens were removed and macerated in DMEM. After centrifugation, the cells were treated with hemolytic ACK buffer at 37 °C for 5 min. The splenocytes were washed twice with PBS, resuspended in DMEM supplemented with 10% FBS, and seeded into 96-well plates (100 µL, 1.0 × 10^5^ cells per well) in the presence of compounds at a range of concentrations (100 μM or 100–1 μM). After incubation for 48 h at 37 °C in 5% CO_2_, cell viability was determined after the addition of 20 µL per well of a solution containing MTT salt (3.0 mM) and phenazine metasulphate (0.72 mM). After further incubation for 1 h at 37 °C, the medium was removed, and DMSO was added to dissolve the dark-blue formazan crystals formed by MTT reduction. Absorbance was measured on a microplate reader at 570 nm [37,41].

### 2.15. Statistical Analysis

The percent cell or parasite viability was calculated by the formula: % viability = [(A − P)/(N − P)] × 100, A = absorbance of specific well, P = positive control, N = negative control. Determination of half-maximal effective concentrations (EC_50_), calculated by logarithmic regression, and significance analysis (*p* < 0.05), using ANOVA and Dunnett’s test, by GraphPad Prism 7.0 software. The results are expressed as the mean and standard deviation (SD) of three independent experiments.

### 2.16. Spectroscopic Procedure for Binding Studies between HSA and 23

Steady-state fluorescence and circular dichroism (CD) spectra were measured on a Jasco J-815 optical spectrometer employing a Jasco PFD-425S15F thermostatic cuvette holder. All spectra were recorded with appropriate background corrections. For the steady-state fluorescence data, inner filter corrections were performed, following the literature [42].

The steady-state fluorescence measurements were carried out in the 290–450 nm range (λ_exc_ = 280 nm) at 289, 296, 303, 310, and 317 K. The addition of compound **23** to a 3.0 mL HSA solution (1.0 × 10^−5^ M, in PBS) was done manually, achieving final concentrations of 0.17, 0.33, 0.50, 0.66, 0.83, 0.99, 1.15, and 1.32 × 10^−5^ M. To obtain quantitative information on the binding capacity of 23 to HSA, Stern-Volmer, double-logarithmic, van’t Hoff, and Gibbs’ free energy analysis were applied following our previous works [43,44,45].

Competitive binding studies were carried out using site probes for sites I, II, and III, i.e., warfarin, ibuprofen, and digitoxin, respectively. The HSA and site probes were used at a fixed concentration (1.0 × 10^−5^ M), and the fluorescence quenching titration with compound **23** was performed as described previously for the steady-state fluorescence quenching procedure at 310 K.

Time-resolved fluorescence measurements were performed on a model FL920 CD fluorimeter from Edinburgh Instruments equipped with an EPL laser (λ_exc_ = 280 ± 10 nm; pulse of 850 ps with energy of 1.8 µW/pulse; monitoring emission at 340 nm). The time-resolved fluorescence decays of a 3.0 mL HSA solution (1.0 × 10^−5^ M, in PBS) were obtained without and with compound **23** (1.32 × 10^−5^ M).

The CD analysis was carried out using 3.0 mL of HSA solution (1.0 × 10^−6^ M, in PBS) without and with 23 in the proportion 1:10 in the 200–250 nm range at 310 K. The average spectra were obtained from three successive runs and corrected by subtraction of the buffer signal. The secondary structure content was estimated by analysis of the CD spectra using the online server BestSel (Beta Structure Selection http://bestsel.elte.hu/index.php; accessed on 12 March 2022).

Synchronous fluorescence (SF) spectra were obtained in a model Xe900 fluorimeter from Edinburgh Instruments. The SF spectra of 3.0 mL HSA solution (1.0 × 10^−5^ M, in PBS) were obtained without and with compound **23** in the 260–320 nm range for Tyr (Δλ = 15 nm) and 240–320 nm range for Trp (Δλ = 60 nm). The ligand concentration was the same used in the steady-state fluorescence studies at room temperature.

### 2.17. Molecular Docking

All molecular docking studies for this analysis were performed with GOLD (Genetic Optimization for Ligand Docking) version 2021.3.0 [46], and the ChemPLP scoring function was chosen to evaluate the docking poses [47]. The three-dimensional structure of tubulin from bos taurus complexed with colchicine was obtained from the Protein Data Bank (PDB ID = 4O2B at 2.3 Å resolution) (http://www.rcsb.org/pdb/; accessed on 15 February 2022) [48,49]. Only chain A was kept in the crystal structure for docking purposes, while the others were deleted. Water molecules, bound inhibitors and cofactors contained in the PDB file have also been removed. The binding site was defined as all atoms within 10 Å of Cys241, which was used as the reference residue. The structures of the studied compounds were manually built with Spartan for Windows v. 8 software (Wavefunction Inc., Tokyo, Japan). Geometry optimization was performed using the semiempirical method PM6 [50]. The protein–ligand complex with the most favorable scores among the top-scoring complexes was used for further visual inspection. The analysis of intermolecular interactions was performed using PyMOL v. 0.99 for Windows [51], and Discovery Studio 2016 [52].

### 2.18. ADMET Prediction in Silico

The ADMET descriptors were calculated using ADMET Lab 2.0 (https://admetmesh.scbdd.com/service/screening/index; accessed on 8 January 2022), SWISS ADME (http://www.swissadme.ch/index.php; accessed on 8 January 2022) and OSIRIS Property Explorer (http://www.cheminfo.org/Chemistry/Cheminformatics/Property_explorer/index.html; accessed on 8 January 2022) servers [53,54,55].

## 3. Results and Discussion

### 3.1. Chemistry

A novel protocol for the synthesis of 1,2,4-oxadiazoles was discovered accidentally while trying to adapt the methodology described by Chiou & Shine (1989) [56]. The reported methodology did not adapt well to our system, and the reactions took over 8 h under reflux in dry pyridine to complete the cyclization of the 1,2,4-oxadiazole ring (results not shown). In addition to its high toxicity [57], pyridine poses an inconvenience when monitoring the reaction by TLC, as it absorbs strongly at λ = 254 nm (log ε = 5.0) [58], meddling with TLC interpretation under UV_254_ exposure. When trying to accelerate pyridine evaporation from the TLC plate using a heat gun, we observed that the reaction was completed instantly, which inspired us to try a new methodology using silica gel as a solid support.

**Scheme 1:**
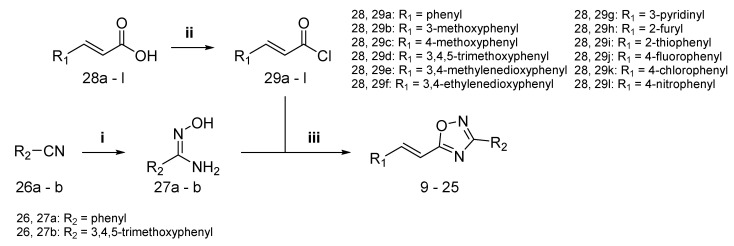
Synthesis of 1,2,4-oxadiazoles **9**–**25**. Reaction conditions: (i) NH_2_OH∙HCl (2.0 eqv), K_2_CO_3_ (1.5 eqv), 8-hydroxyquinoline (0.35 mol %), EtOH/H_2_O 1:1, reflux, 60 min. (ii) (COCl)_2_ (solvent), r.t., 30 min. (iii) benzamidoxime (27), acid chloride 29 (1.2 eqv), K_2_CO_3_ (1.5 eqv), DCM, r.t., 30 min., then silica gel (solvent free), microwave irradiation, 75 W, 105 °C, 5–45 min.

The synthesis of 1,2,4-oxadiazole derivatives **9**–**25** started from the conversion of properly substituted benzonitriles (**26a**–**b**) into benzamidoximes (**27a**–**b**). 3-Aryl-acrylic acids (**28a**–**l**) reacted in the presence of excess oxalyl chloride (solvent) to yield the corresponding acid chlorides (**29a**–**l**), which were used in the next step without further purification after the solvent was removed under vacuum. The next step was conducted with dry solvents, reagents, and glassware to avoid hydrolysis of acid chlorides. 3-Aryl-acryloyl chlorides (**29a**–**l**) were fully dissolved in dry DCM and added dropwise over benzamidoximes (**27a**–**b**) in the presence of the base. After the nucleophilic acyl substitution reaction was completed, silica gel was added, and the solvent was completely removed. The cyclization step was conducted under microwave irradiation for a better heat distribution over the reaction [59]. Scheme 1 depicts the synthesis of 1,2,4-oxadiazole derivatives **9**–**25**, and the reaction conditions are captioned.

The novel methodology allowed us to obtain compounds **9**–**25** with yields ranging between 50–80%. The cyclization step assisted by microwave irradiation required different reaction times depending on the R_1_ and R_2_ groups. The detailed reaction yields and times required for the cyclization step are reported in Table 1.

**Scheme 2:**
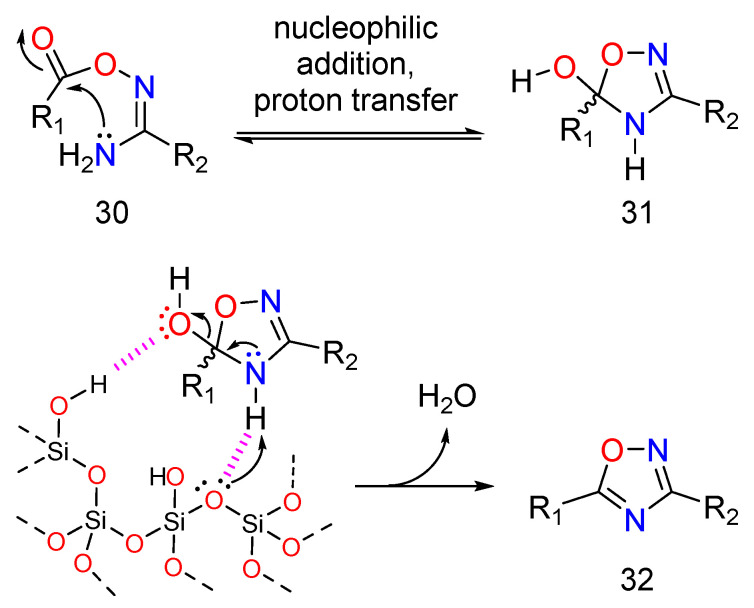
Proposed mechanism for the cyclization reaction in the presence of silica gel to yield 1,2,4-oxadiazoles.

The mechanism for the cyclization step is proposed as a 1-5 intramolecular nucleophilic attack from the amino group to the carboxyl carbon in the *O*-acyl-amidoxime intermediate (30). The resulting 4,5-dihydro-1,2,4-oxadiazol-5-ol intermediate (31) should then eliminate water to become the more stable 1,2,4-oxadiazole (32). Silica gel is proposed to act by diminishing the energy barrier for the dehydration reaction by interacting with 31 via hydrogen bond acceptance and donation involving silanol and silanoxide groups, as shown in Scheme 2.

### 3.2. Biological Assessment

#### 3.2.1. Antiproliferative Activity against Drug-Resistant Leukemia Cell Lines

Compounds **9**–**25** were evaluated against the CML cell lines K562, Lucena-1 (K562/VCR), and FEPS (K562/DNR). The MDR phenotype was induced in vitro by exposing K562 cells (ATCC) to increasing concentrations of the antimitotic drug vincristine sulfate (Lucena-1, VCR) or the topoisomerase inhibitor daunorubicin hydrochloride (FEPS, DNR) [35,36]. In most cases, compounds **9**–**25** showed similar or higher activity over drug-resistant cells compared with wild-type K562 cells. MDR cells overexpress ATP-binding cassette (ABC) efflux transporters, which are responsible for extruding a vast variety of amphiphilic compounds, thus reducing the cytoplasmic concentration of drugs [60].

Compounds **12**, **15**, **23**, and **24** were able to inhibit cell growth to 50% at concentrations below 20 μM, with derivative **23** being the most active against all cell lines. Notably, its structure shows the (*E*)-vinyl-1,2,4-oxadiazole motif bearing two 3,4,5-trymethoxy-phenyl groups, an antiproliferative pharmacophore present on known antitubullin agents [61]. Table 2 contains the calculated values of effective concentrations in 50% over cell growth (EC_50_) for compounds **9**–**25** against K562, Lucena-1, and FEPS cell lines.

The relative resistance (RR) index was calculated as the ratio between the EC_50_ values for MDR and parental cells. Values of RR > 2.00 indicate a drug resistance profile, whereas RR < 0.50 indicate a collateral sensitivity (CS) profile. Although the mechanisms associated with CS are not completely understood, they are believed to be influenced by ATP depletion after futile cycling during ABC transporter-mediated drug efflux, ABCC1-mediated extrusion of glutathione, elevated levels of ROS production after redox cycling, alteration of drug target proteins, and impairment of plasma membrane fluidity [62,63]. Aside from compounds **9**, **10**, **12**, **16**, and **20**, all oxadiazoles showed higher or equivalent toxicity to one or both MDR cells in comparison to the parental lineage (Table 2). Compounds **13** (RR = 0.50, Lucena-1), **23** (RR = 0.42, FEPS), and **24** (RR = 0.15, FEPS) showed the sharpest CS profiles within the series of compounds.

#### 3.2.2. Antitrypanosomal Activity

The activity against trypanosome parasites was evaluated against *T. cruzi* and *L. amazonensis*. Despite the differences between the species, trypanosomatids share similarities and common targets that can be explored for the development of antiparasitic drugs [65,66]. The antitrypanosomal activity was evaluated against *T. cruzi* amastigotes expressing the beta-galactosidase gene (Tulahuen C2C4 LacZ strain) [37] infected in LLC-MK2 fibroblasts. Compounds **9**–**25** were added to the cell culture at 50 μM, and parasites were cultured for 120 h before being indirectly quantified by cleavage of the substrate chlorophenol-red-β-galactopiranoside (CPRG). The reference drug benznidazole was also evaluated by means of comparison. The toxicity against LLC-MK2 host cells at the same concentrations was determined using the MTT method [39]. Leishmanicidal activity was evaluated on *L. amazonensis* (Josefa strain) against promastigotes. Parasites were cultured for 48 h in the presence of compounds **9**–**25** at serially diluted concentrations. The EC_50_ values were calculated and compared with the reference drug amphotericin B. The results for the initial activity screening against trypanosomatids are reported in Table 3.

Four compounds were able to reduce the *T. cruzi* amastigote percentage below 50% compared with untreated controls, **12** (37.8%), **15** (33.8%), **23** (2.9%), and **24** (42.2%), which were selected for EC_50_ determination against *T. cruzi* amastigotes and LLC-MK2. The three compounds with the lowest EC_50_ values against *L. amazonensis* promastigotes (**12**, 26.0 μM; **23**, 12.2 μM; **24**, 10.0 μM) were also selected for evaluation in RAW 264.7 murine macrophages infected with intracellular amastigotes. For this experiment, host cells were lysed with 0.01% Triton-X 48 h after treatment, and parasites were allowed to differentiate into promastigotes for 48 h before determination of viability by the MTT method [39,40]. The toxicity to RAW 264.7 cells was also determined. Table 4 contains the EC_50_ results against *T. cruzi* and *L. amazonensis* intracellular amastigotes and against LLC-MK2 and RAW 264.7 host cells.

**Figure 3:**
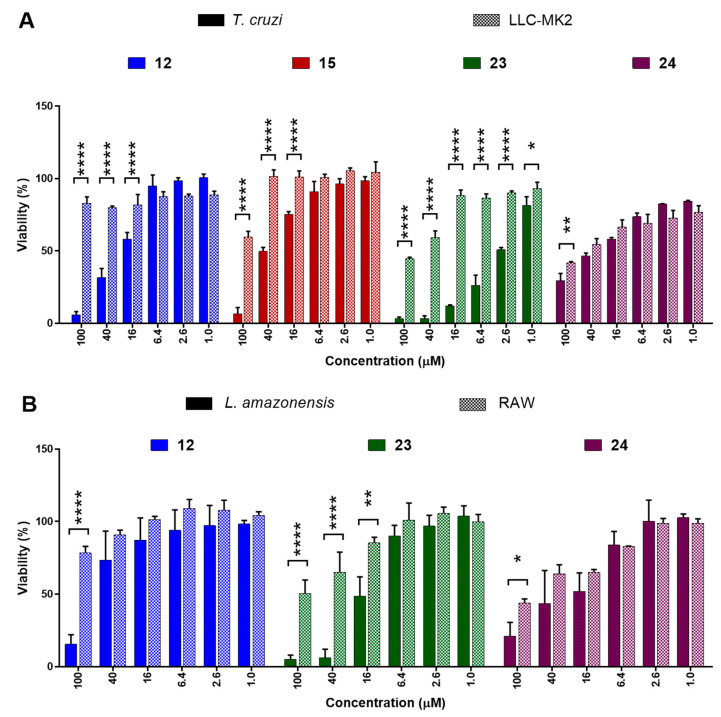
Comparison between the effects of treatment with 1,2,4-oxadiazoles against intracellular amastigote and host cell growth. (**A**) Compounds **14**, **17**, **25**, and **26** against *T. cruzi* and LLC-MK2 cells. (**B**) Compounds **14**, **25**, and **26** against *L. amazonensis* and RAW 264.7 cells. * = *p* < 0.05; ** = *p* < 0.01; **** = *p* < 0.0001.

The 1,2,4-oxadiazoles evaluated against trypanosomatids were able to selectively inhibit parasite growth of intracellular amastigotes. Compound **23**, which was the most active against both *T. cruzi* and *L. amazonensis*, was able to significantly inhibit amastigote growth (81.3 ± 6.2% viability) compared with host cell toxicity (93.0 ± 4.4% viability) at concentrations as low as 1.0 μM for *T. cruzi* and 16 μM for *L. amazonensis* (*p* < 0.05). The comparative plots containing the experimental data for antitrypanosomatid (intracellular amastigotes) and cytotoxic activities (host cells) after treatment with compounds **12**, **15**, **23**, and **24** are depicted in Figure 3.

#### 3.2.3. Toxicity to Murine Splenocytes

**Figure 4:**
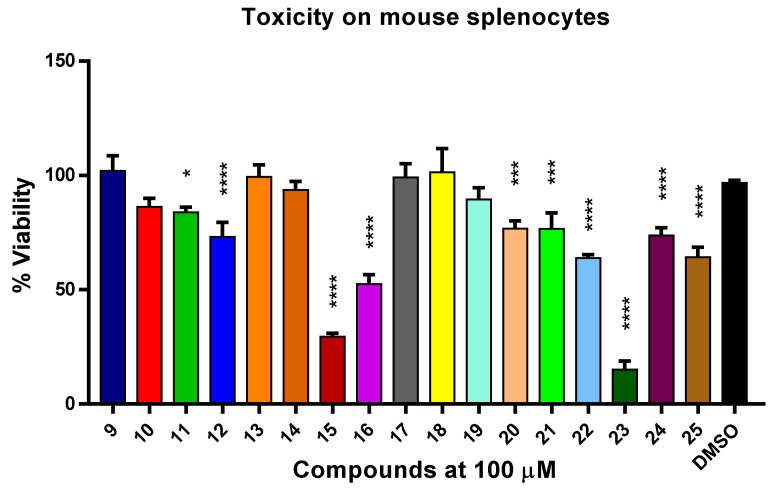
Toxicity of compounds **9**–**25** to murine splenocytes (BALB/c mice). Statistical significance was determined in comparison to the untreated control. * = *p* < 0.05; *** = *p* < 0.001; **** = *p* < 0.0001.

To assess safety, the toxicity of compounds was also determined against primary murine splenocytes harvested from BALB/c mice. 1,2,4-Oxadiazoles **9**–**25** were treated at 100 µM for 48 h, and the evaluation of mitochondrial activity was determined by the MTT method [37,41]. The compounds displayed diverse profiles of reduced splenocyte viability in vitro, as displayed in Figure 4 (actual viability values can be found in Table S1). Compounds **15** and **23** reduced splenocyte viability to less than 50%. They were selected for determination of EC_50_, which returned 52.18 ± 4.51 µM for 15 and 33.30 ± 2.83 µM for 23 (Table S2). Diverse cell subpopulations from the immune system are resident on the spleen, mostly T and B lymphocytes, but myeloid cells such as macrophages are also present [67]. Of note, regardless of being more active, compounds **15** and **23**, along with **12** and **24**, showed selective profiles of toxicity to chemotherapy refractory leukemias and to parasitic protozoa.

### 3.3. Theoretical Studies in Silico

#### 3.3.1. Molecular Docking with Bovine Tubulin

**Figure 5:**
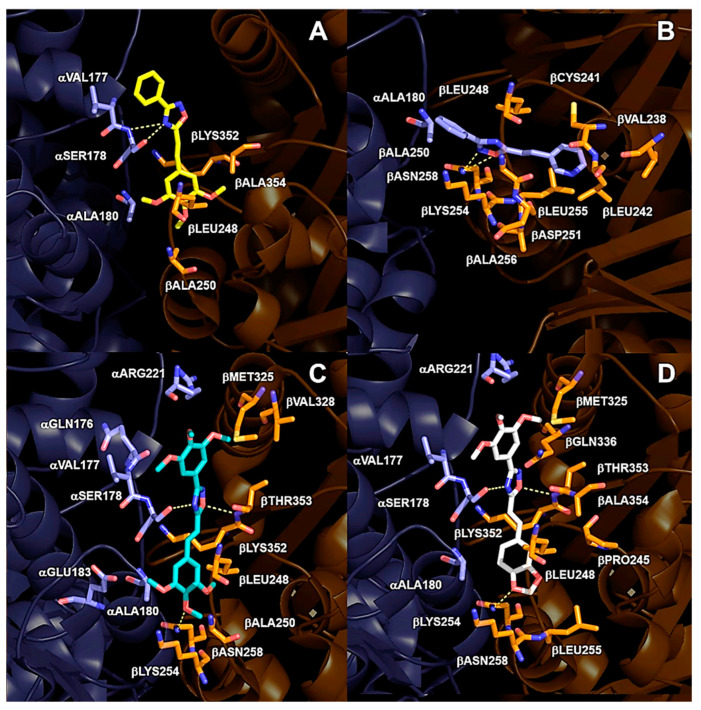
Best docking poses at CBS (α and β monomers of bovine tubulin in purple and orange, respectively) obtained for compounds **12** (**A**, yellow carbon atoms), **15** (**B**, purple carbon atoms), **23** (**C**, cyan carbon atoms), and **24** (**D**, gray carbon atoms). Hydrogen bonds are in dashed yellow lines. Hydrogen atoms have been omitted for better visualization.

Docking simulations were performed to gain some insight into the putative binding modes of representative compounds **12**, **15**, **23**, and **24** with the colchicine binding site (CBS) of tubulin. This site was selected for evaluation due to the presence of the 3,4,5-trimethoxyphenyl moiety on compounds **12**, **23**, and **24** as much as on colchicine. Tubulin is involved in spindle formation during mitosis. It is a polymeric protein composed of two different isoforms, alpha- and beta-tubulin, that act as monomers, alternating throughout its quaternary structure. The top poses obtained in the process are depicted in Figure 5.

Analysis of the docking pose of 12 and tubulin (Figure 5A) showed two hydrogen bonds involving N4 of the oxadiazole ring, the OH group of the side chain of αSER178, and the NH group of the peptide backbone of this amino acid. Additionally, an interaction of the pi-alkyl type involving the 3,4,5-methoxy phenyl ring and the side chain of βLEU248 was observed. Interactions of the alkyl type were suggested with αALA180, βLEU248, βALA250, βLYS352, and βALA354 and involved the carbon atoms of the side chains of the amino acids and the methyl groups in the 3,4,5-methoxy phenyl ring. Finally, αVAL177, βLYS352 and the 3,4,5-methoxy phenyl ring were involved in pi-alkyl interactions.

Visual inspection of the docking pose of 15 and tubulin (Figure 5B) showed hydrogen bonding between the oxygen atom and N2 of the oxadiazole ring with the amino group of the side chain of βASN258. Carbon hydrogen bonds were also observed and involved the oxygen atom of the peptide carbonyl of βVAL238 and βASP251 with hydrogen atoms in the pyridine ring. In addition, pi-sigma interactions were observed, involving the side chain of βLEU255 and the hydrocarbon spacer of the compound. Finally, a few pi-alkyl interactions were observed, involving the amino acid residues αALA180 and βLYS254 with the phenyl ring, βCYS241 and βLEU242 with the pyridine ring, and βLEU248 and βALA250 with the oxadiazole ring.

The analysis of the docking pose of 23 and tubulin (Figure 5C) showed hydrogen bonds involving N4 of the oxadiazole ring and αSER178, the oxygen atom of this ring and the amino group of the peptide backbone of βTHR353, and the amino group of the side chain of βASN258 and the oxygen atom of the 4-methoxy phenyl moiety. Furthermore, carbon hydrogen bonds were suggested, involving amino acid residues αGLN176, αSER178, αGLU183 and βMET325 and the molecular skeleton of the compound. Alkyl interactions were also observed and involved αVAL177, αALA180, αARG221, βALA250, βLYS254, βMET325, βVAL328 and βLYS352 and the hydrocarbon skeleton of 23. Finally, pi-alkyl interactions of αALA180, βLEU248 and βLYS352 with the 3,4,5-methoxy phenyl ring were also observed.

Visual inspection of compound **24** and tubulin (Figure 5D) showed hydrogen bonding involving N4 of the oxadiazole ring and αSER178, the oxygen atom of the oxadiazole ring and the peptide NH group of βTHR353 and, finally, a hydrogen bond involving one of the oxygen atoms of the 1,3-benzodioxole ring and the amino group of the side chain of αASN258. Carbon hydrogen bonds were suggested with αARG221, βLYS254, and βMET325. Other observed interactions were of the alkyl type and involved αVAL177, αARG221 and βLEU255 and βMET325. Finally, pi-alkyl interactions involving αALA180 and βLEU248 with the 1,3-benzodioxole ring and βLYS352 and the oxadiazole ring were suggested.

A compilation of the main interacting amino acid residues of CBS belonging to the α and β monomers observed by docking is presented in Table 5. Comparison with the binding environment of colchicine in α and β monomers shows that **15** interacts in a closer vicinity compared with the colchicine binding environment, although the interaction types may differ. This does not come as a surprise since this compound does not bear methoxy substituents and can fit better in CBS in its fairly extended and rigid shape. In addition, **23** and **24** share a similar binding orientation in CBS, while **12** stands in the middle.

**Figure 6:**
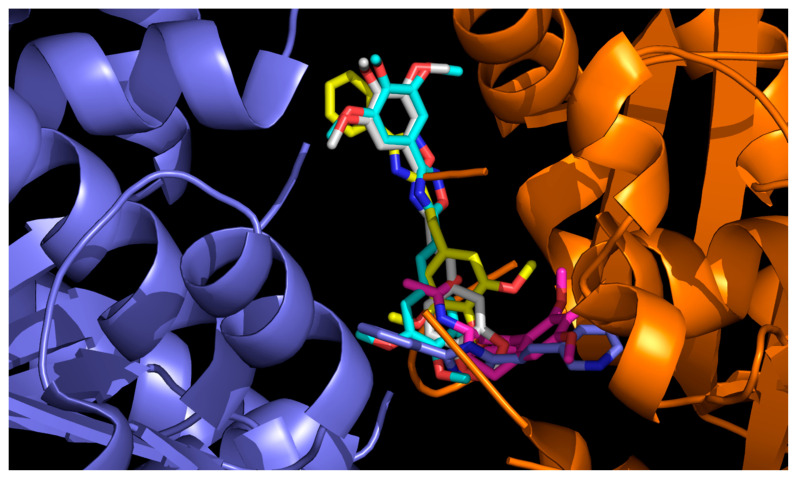
Best docking poses at CBS (α and β monomers of bovine tubulin in purple and orange, respectively). Colchicine (magenta), **12** (yellow), **15** (purple), **23** (cyan), and **24** (gray).

The superimposition of the representative compounds with colchicine in the crystal structure (Figure 6) highlights that compounds **12**, **23**, and **24** are localized at the dimer interface better than at zone 2 of CBS.

Due to steric restraints, related to the mostly planar and rigid nature of the compounds, they are not oriented toward the most buried (and narrower) region of CBS, although they may interact with amino acid residues of the β monomer closer to the dimer interface. In particular, compound **23**, the most active compound in the series, is clearly surrounded by more amino acid residues from the α monomer compared with the other compounds, which may account for its better theoretical affinity to tubulin.

#### 3.3.2. ADMET Predictions and Experimental Interaction with Human Serum Albumin (HSA)

Ideally, in addition to its high potency and specificity, a drug should possess a set of characteristics that favor its absorption, distribution, metabolization, and excretion from the body; at the same time, it displays reduced toxicity. Proper ADMET prediction at the early stage of drug development can prevent pharmacokinetics- and toxicity-related drawbacks in clinical stages. In this regard, Lipinski’s rule of five is an important set of descriptors concerning ADME properties that take into consideration the molecular weight (MW < 500 Da), number of hydrogen bond (HB) donors (<5), and acceptors (<10) and lipophilicity (LogP < 5) [68]. Some other key parameters taken into consideration when evaluating druglikeness are the topological polar surface area (TPSA < 140 Å^2^) and the number of rotatable bonds (RB < 11) present in the molecule [69]. The physicochemical, pharmacological, and toxicological properties of compounds **9**–**25** were predicted in silico and compared with the reference drugs benznidazole, amphotericin B, and colchicine. For compounds **9**–**25**, as well as for benznidazole and colchicine, Lipinski’s rule was not violated, and optimal TPSA and RB values were found. The antileishmanial drug amphotericin B failed Lipinski’s test, and a nonoptimal TPSA value was found. The calculated physicochemical properties for compounds **9**–**25** and the reference drugs benznidazole, amphotericin B, and colchicine are presented in Table 6.

All compounds, **9**–**25**, are predicted to have high human intestinal absorption (HIA). This result was also observed for reference drugs, apart from amphotericin B, which is administered intravenously. General toxicity was also assessed concerning mutagenic, tumorigenic, skin irritant, and reproductive risks. Compounds **13** and **24** were found to be potentially toxic due to reproductive risks because of the piperonyl group [70], whereas **16** and **25** were found to be potentially tumorigenic due to the presence of the 2-furoyl group [71]. Affinity to human P-glycoprotein (P-gp), an ABC efflux transporter from subfamily B (ABCB1) [72], was also predicted. Compounds **9**–**25** showed a diverse behavior toward P-gp between substrate, inhibitor, or no interaction. Interestingly, the most active compounds against MDR leukemias, **23** and **24**, showed no interaction with the P-gp efflux pump. Finally, a fragment-based assessment was also carried out to determine the drug likeness (DL) of compounds **9**–**25** and compared them to reference drugs. The drug score (DS), which considers drug likeness, cLogP, logS, molecular weight, and toxicity risks, was also calculated. Based on such predictions, compounds **9**–**25** displayed good druggability (DS = 0.6–0.9), with a high potential for oral administration and a favorable ADMET profile. Compounds **13**, **14**, **16**, **20**, **24**, and **25** showed reduced druggability (DS < 0.6), mainly because of low drug likeness or toxicity risks. A summary of the pharmacokinetic and toxicity analysis results is presented in Table 7.

**Figure 7:**
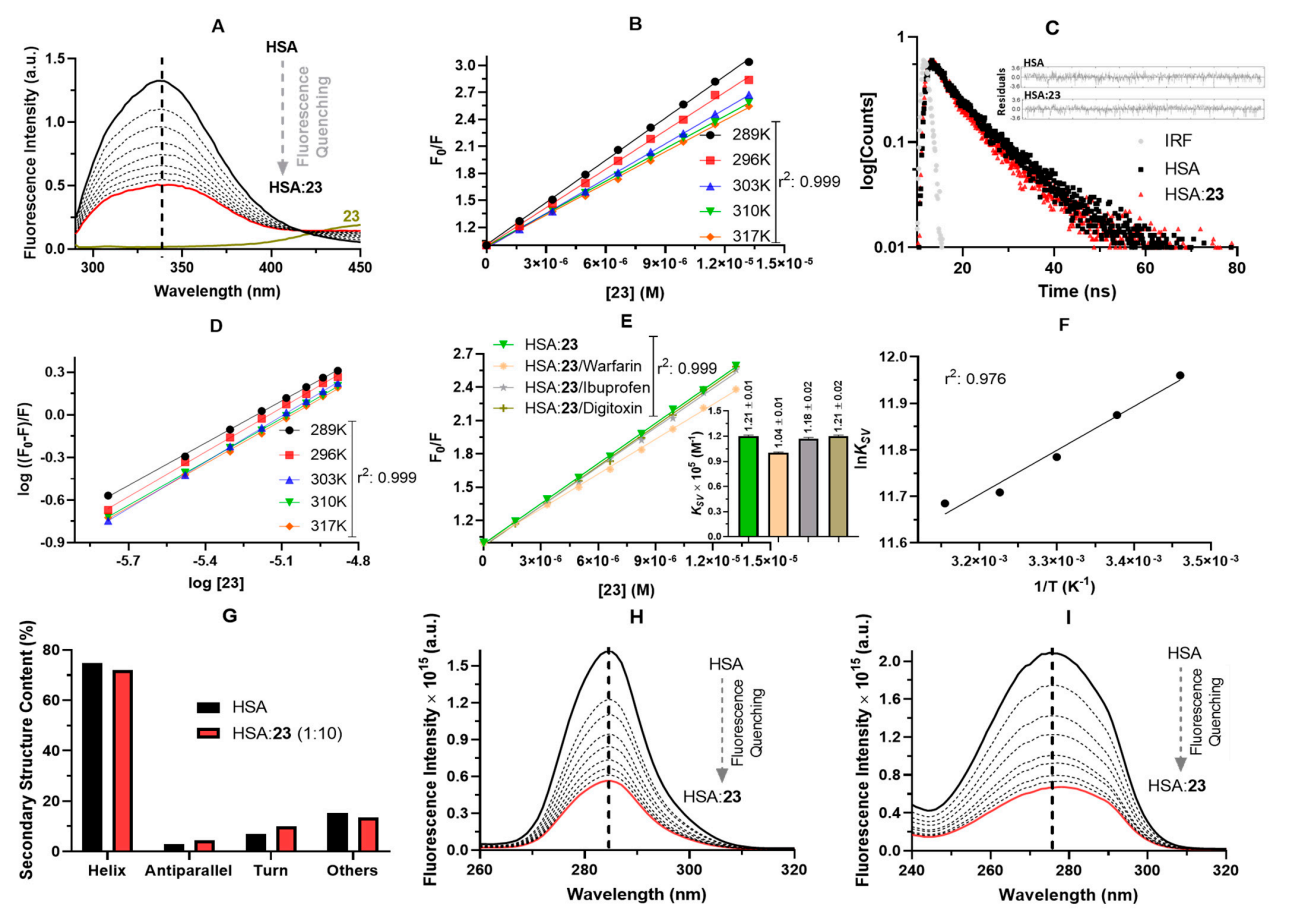
(**A**) Steady-state fluorescence emission spectra (λ_exc_ = 280 nm) for HSA without and upon successive additions of 23 at pH = 7.4 and 310 K. (**B**) Stern-Volmer plots for the HSA:23 interaction in PBS. (**C**) Time-resolved fluorescence decays for HSA (1.0 × 10^−5^ M) without and with 23 (1.32 × 10^−5^ M) at pH = 7.4 and 296 K. The residuals were obtained by biexponential treatment with χ^2^ values of 1.027 and 1.142 for HSA and HSA:23, respectively. (**D**) Double-logarithmic plots for HSA:23 in PBS. (**E**) Stern-Volmer plots for HSA:23 without and with the site markers warfarin, ibuprofen, or digitoxin at 310 K. (**F**) Van’t Hoff plot based on *K_SV_* values for HSA:23. (**G**) Secondary structure content of HSA (1.0 × 10^−6^ M) without and with 23 (proportion 1:10). SF spectra of HSA without and upon successive additions of 23 at (**H**) Δλ = 15 nm for Tyr residues and (**I**) Δλ = 60 nm for Trp residues in PBS at room temperature. Concentrations: HSA = 1.0 × 10^−5^ M; 23 = 0.17, 0.33, 0.50, 0.66, 0.83, 0.99, 1.15, and 1.32 × 10^−5^ M.

Among the (*E*)-3-aryl-5-(2-aryl-vinyl)-1,2,4-oxadiazoles derivatives that presented the best antiparasitic and anticancer profile, compound **23** was selected as a model to study the interactions with HSA—the main globular protein in the human bloodstream, which is responsible for the distribution of several endogenous and exogenous molecules [73]—to complement the ADMET predictions. The successive addition of 23 into the HSA solution resulted in a slight bathochromic shift (±5 nm) in the steady-state fluorescence emission of albumin (Figure 7A), indicating that compound **23** might perturb the microenvironment around the fluorophores Trp or Tyr residues [42]. The Stern-Volmer plots (Figure 7B) are linear, with Stern-Volmer quenching constant (*K_SV_*) values decreasing with increasing temperature (Table 8). The bimolecular quenching rate constant (*k_q_*) values are on the order of 10^13^ M^−1^s^−1^ (Table 8) and are larger than the maximum diffusion rate constant in water (*k_diff_* ≈ 7.40 × 10^9^ M^−1^s^−1^ at 298 K, according to Smoluchowski-Stokes-Einstein theory at 298 K) [74]. These observations indicate a ground-state association between HSA and 23 (static fluorescence quenching mechanism), which was further confirmed by the superposition of the time-resolved fluorescence decays of HSA and HSA:23 (Figure 7C) with the same quantitative parameters inside the experimental error (Table 8) [43,44].

For a purely static fluorescence quenching mechanism, the *K_SV_* values also estimate the binding affinity [42,44], and for this reason, the double-logarithmic approximation (Figure 7D) was applied only to determine the number of binding sites (*n*). The *K_SV_* values on the order of 10^5^ M^−1^ (Table 8) indicate a moderate binding affinity, which is higher than the reported constant for HSA with 1,3,4-oxadiazole [75] and 1,3,4-oxadiazolyl-1,2,4-oxadiazoles [45] derivatives. Moderate binding affinities of bioactive substances to serum albumin implicate favorable absorption and distribution profiles. Hence, the absorption and distribution of compound **23** to the target can be considered feasible [73]. This conclusion is also consistent with the in silico predictions that suggested compound **23** does not interact with P-gp (Table 7), given this transporter is abundantly present in the intestinal epithelium, pumping drugs back into the lumen and decreasing their absorption [76]. The *n* values in the range of 0.992–1.09 indicate an interaction in the proportion 1:1—one single albumin molecule binds with one molecule of 23 (Table 8). The main binding site for the studied compound is subdomain IIA (site I), where the Trp-214 residue can be found (the *K_SV_* value decreased 14% in the presence of warfarin, Figure 7E), in accordance with the static fluorescence quenching mechanism previously described above [44,73].

According to Ross and Subramanian’s analysis [77], the sign and magnitude of the thermodynamic parameters, i.e., enthalpy (Δ*H*°) and entropy (Δ*S*°), changes are important to determine the interaction forces involved in protein-ligand interactions. In this sense, van’t Hoff analysis was carried out (Figure 7F), and the obtained Δ*S*° > 0 (Table 8) can be related to hydrophobic forces or the desolvation process, while Δ*H*° < 0 (Table 8) can be related to electrostatic forces, including van der Waals interactions. Therefore, further the spontaneity (Δ*G*° < 0, Table 8), the association HSA:23 is entropically and enthalpically driven [43]. Finally, the structural analysis from circular dichroism (CD) and synchronous fluorescence (SF) approaches shows that 23 does not significantly perturb both the secondary content of HSA (Figure 7G) and the microenvironment around Tyr residues (Figure 7H) [74]. However, it is important to highlight that SF data at Δλ = 60 nm (bathochromic shift, Figure 7I) identified perturbation of the microenvironment around the Trp residue upon binding of 23, agreeing with the steady-state fluorescence quenching data (Figure 7A). This perturbation is probably due to the desolvation effect (increase in the hydrophobicity inside subdomain IIA) as previously detected by thermodynamic analysis on the nature of the binding.

## 4. Conclusions

The novel method employed for the synthesis of the 1,2,4-oxadiazole ring was efficient for the preparation of compounds **9**–**25**. The simple experimental, quick reaction times and yields are factors that make this protocol a potentially useful tool for the synthesis of 1,2,4-oxadiazoles for different applications. The strategy of using the (*E*)-3-aryl-5-(2-aryl-vinyl)-1,2,4-oxadiazole motif as a scaffold for the search for antiproliferative agents has proven promising. Compounds were able to inhibit the growth of human leukemias and selectively target intracellular amastigotes of *T. cruzi* and *L. amazonensis*. Compound **23** displayed the lowest EC_50_ values against all cell types investigated (K652, 13.2 µM; Lucena-1, 12.4 µM; FEPS, 5.5 µM; *T. cruzi*, 2.9 µM; *L. amazonensis*, 13.5 µM) and had a good binding profile with HSA. The presence of the antimitotic pharmacophore 3,4,5-trimethoxy-phenyl is critical for the activity of compounds. These results have been corroborated by docking studies at the colchicine binding site of human tubulin. The pharmacokinetic predictions have a favorable drug-likeness profile and are consistent with the good binding profile with HSA that has been observed for hit compound **23**. However, the in vitro assessment of mouse splenocytes highlights the need for further structural optimization to reduce toxicity while maintaining favorable antiproliferative activities. In conclusion, we found evidence that the (*E*)-5-(vinyl)-1,2,4-oxadiazole motif is compatible with molecular hybridization as a drug design strategy and is a promising scaffold for the development of new antiproliferative agents.

## Figures and Tables

**Table 1:** Reaction times and yields for the synthesis of compounds **9**–**25**.

Compound	Structure	MW Time (min) ^a^	Yield (%)
**9**	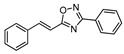	10	68
**10**	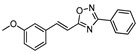	5	75
**11**	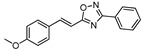	5	60
**12**	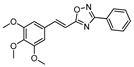	5	70
**13**	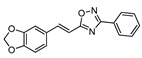	5	64
**14**	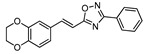	10	70
**15**	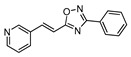	15	68
**16**	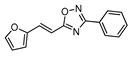	10	61
**17**	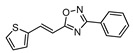	5	57
**18**	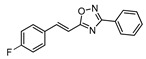	15	55
**19**	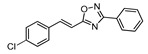	15	50
**20**	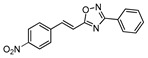	45	79
**21**	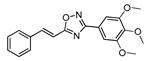	10	73
**22**	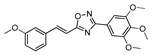	5	60
**23**	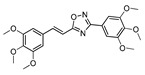	5	76
**24**	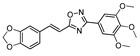	10	60
**25**	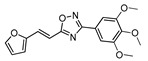	15	61

^a^ Reaction times under microwave irradiation for the synthesis of the 1,2,4-oxadiazole ring.

**Table 2:** Antiproliferative activity of compounds **9**–**25** against drug-resistant CML cell lines.

Compound	EC_50_ (μM)				
	K562	Lucena-1	RR ^a^	FEPS	RR ^a^
**9**	>100	>100	-	>100	-
**10**	>100	>100	-	>100	-
**11**	>100	>100	-	58.8 ± 6.1	<0.59
**12**	18.0 ± 0.9	43.5 ± 1.8	2.42	25.5 ± 4.9	1.42
**13**	92.3 ± 4.2	46.5 ± 1.9	0.50	68.5 ± 8.7	0.74
**14**	>100	64.0 ± 3.2	<0.64	41.1 ± 4.9	<0.41
**15**	27.6 ± 5.8	26.7 ± 7.2	0.97	18.7 ± 3.9	0.68
**16**	52.8 ± 17.7	65.0 ± 9.6	1.23	54.7 ± 1.7	1.03
**17**	>100	71.9 ± 0.6	<0.72	55.7 ± 17.2	<0.56
**18**	>100	59.9 ± 12.2	<0.59	66.7 ± 9.6	<0.67
**19**	47.1 ± 1.2	43.4 ± 3.4	0.92	>100	>2.12
**20**	34.4 ± 7.4	45.7 ± 4.3	1.33	46.4 ± 4.9	1.35
**21**	>100	68.6 ± 10.1	<0.69	27.7 ± 5.2	<0.28
**22**	>100	72.9 ± 3.0	<0.73	36.1 ± 12.4	<0.36
**23**	13.2 ± 2.9	12.4 ± 2.5	0.94	5.5 ± 0.6	0.42
**24**	60.4 ± 4.1	82.6 ± 10.8	1.37	8.9 ± 0.3	0.15
**25**	72.8 ± 9.1	76.6 ± 6.1	1.05	53.7 ± 3.6	0.74
VCR ^b^	0.054 ± 0.005	0.85 ± 0.12	15.7	1.05 ± 0.07	19.4
DNR ^b^	0.082 ± 0.001	3.06 ± 0.87	37.3	6.45 ± 1.27	78.6

^a^ Relative resistance (RR): (EC_50_ MDR cells)/(EC_50_ parental cells) ratio. ^b^ VCR = vincristine sulfate; DNR = daunorubicin hydrochloride, EC_50_ values from reference [64].

**Table 3:** Screening results against *T. cruzi* amastigotes and LLC-MK2 host cells at 50 μM and EC_50_ on *L. amazonensis* promastigotes.

Compound	Screening at 50 μM		*L. amazonensis* Promastigotes
	*T. cruzi* Amastigotes	LLC-MK2	
	% Viability	% Viability	EC_50_ (μM)
**9**	87.0 ± 4.9	87.2 ± 4.0	74.5 ± 4.9
**10**	77.7 ± 6.3	86.6 ± 3.6	50.8 ± 6.3
**11**	86.2 ± 5.3	77.4 ± 4.0	>100
**12**	37.8 ± 2.6	88.2 ± 5.2	26.0 ± 3.0
**13**	72.0 ± 3.7	75.9 ± 1.3	>100
**14**	60.5 ± 3.8	84.5 ± 4.6	92.5 ± 3.8
**15**	21.5 ± 2.2	70,0 ± 4.8	53.6 ± 2.2
**16**	83.0 ± 3.7	89.2 ± 1.4	47.1 ± 3.7
**17**	69.6 ± 6.3	86.2 ± 7.1	77.6 ± 6.3
**18**	82.8 ± 4.7	90.7 ± 4.3	>100
**19**	83.4 ± 5.9	77.8 ± 0.9	>100
**20**	74.5 ± 5.4	78.2 ± 2.1	>100
**21**	77.3 ± 2.0	88.5 ± 4.7	>100
**22**	66.1 ± 2.3	87.1 ± 1.7	>100
**23**	8.6 ± 1.2	64.1 ± 4.4	12.2 ± 0.7
**24**	42.2 ± 2.7	55.9 ± 9.3	10.0 ± 2.7
**25**	79.5 ± 5.5	73.5 ± 1.7	>100
Benznidazole	23.2 ± 1.4	-	-
Amphotericin B	-	-	0.094 ± 0.001

**Table 4:** Evaluation of compounds **12**, **15**, **23**, and **24** against intracellular amastigotes of *T. cruzi* and *L. amazonensis* and LLC-MK2 and RAW host cells.

Compound	EC_50_ (μM) ^a^		S.I. ^b^	EC_50_ (μM) ^a^		S.I. ^b^
	*T. cruzi*	LLC-MK2		*L. amazonensis*	RAW	
**12**	22.5 ± 3.7	>100	>4.5	50.1 ± 16.4	>100	>2.0
**15**	33.8 ± 2.3	>100	>3.0	NT ^c^	NT ^c^	-
**23**	2.9 ± 0.2	78.0 ± 4.7	26.7	13.5 ± 3.6	>100	>7.4
**24**	28.7 ± 3.1	53.4 ± 2.6	1.9	24.8 ± 10.4	58.8 ± 6.1	2.3
Benznidazole	1.5 ± 0.2	>100	>69	-	-	-
Amphotericin B	-	-	-	0.151 ± 0.022	9.8 ± 3.3	65.2

^a^ The comparison between EC_50_ values to amastigotes and the respective host cells is statistically significant (*p* < 0.001 in all cases) ^b^ Selectivity index (SI): host cell/parasite EC_50_ ratio. ^c^ Not tested.

**Table 5:** Comparison of the main interacting residues of representative compounds observed by molecular docking and the binding mode of colchicine in the crystal structure of tubulin dimer.

Compound	Main Interacting Residues ^a^	
	α Monomer	β Monomer
Colchicine ^b^	ALA180, VAL181	VAL238, CYS241, LEU242, LEU248, ALA250, ASP251, LEU255, MET259, VAL315, ALA316, ALA317, ILE318, ASN350, LYS352, ALA354
**12**	SER178, VAL177, **ALA180**	**LEU248**, **ALA250**, **LYS352**, **ALA354**
**15**	**ALA180**	**VAL238, CYS241, LEU242, LEU248, ALA250, ASP251**, LYS254, **LEU255**, ASN258
**23**	GLN176, VAL177, SER178, **ALA180**, GLU183, ARG221	**LEU248, ALA250**, LYS254, ASN258, MET325, **VAL328**, **LYS352**, THR353
**24**	VAL177, SER178, **ALA180**, ARG221	**LEU248**, LYS254, **LEU255**, ASN258, MET325, **LYS352**, THR353

^a^ Interacting residues in common with colchicine are in bold type. ^b^ Interactions observed in the crystal structure. The bold format indicates the amino acid residues that interact both with colchicine and a given compound.

**Table 6:** Physicochemical parameters for compounds **9**–**25** and reference drugs benznidazole, amphotericin B, and colchicine.

Compound	MW ^a^ (g/mol)	cLogP	TPSA ^b^ (Å^2^)	Rotatable Bonds	HB ^c^ Acceptors
**9**	248.28	3.60	38.92	3	3
**10**	278.31	3.55	48.15	4	4
**11**	278.31	3.55	48.15	4	4
**12**	338.36	3.52	66.61	6	6
**13**	292.29	3.39	57.38	3	5
**14**	306.32	3.42	57.38	3	5
**15**	249.27	2.81	51.81	3	4
**16**	238.25	2.89	52.06	3	4
**17**	254.31	3.55	67.16	3	3
**18**	266.27	3.87	75.06	3	4
**19**	282.73	4.09	38.92	3	3
**20**	293.28	2.95	84.74	4	5
**21**	338.36	3.60	66.61	6	6
**22**	368.39	3.53	75.84	7	7
**23**	428.44	3.50	94.30	**9**	**9**
**24**	382.37	3.46	85.07	6	8
**25**	328.32	2.94	86.85	6	7
Benznidazole	260.25	0.49	98.34	6	4
Amphotericin B	924.09	0.32	319.61	3	18
Colchicine	399.44	2.36	83.09	6	6

^a^ Molecular weight. ^b^ Topological polar surface area. ^c^ Hydrogen bond.

**Table 7:** Pharmacokinetic and toxicity in silico prediction of compounds **9**–**25** and reference drugs.

Compound	HIA ^a^	Toxicity Risk ^b^	P-gp ^c^	DL ^d^	DS ^e^
**9**	High	None	I, S	0.65	0.62
**10**	High	None	S	1.80	0.81
**11**	High	None	S	2.20	0.83
**12**	High	None	S	5.50	0.85
**13**	High	Reproductive	S	2.34	0.49
**14**	High	None	S	−4.68	0.43
**15**	High	None	I, S	2.59	0.9
**16**	High	Mutagenic	I, S	1.89	0.52
**17**	High	None	I, S	3.78	0.87
**18**	High	None	S	1.09	0.75
**19**	High	None	I, S	2.83	0.78
**20**	High	None	I, S	-8.32	0.46
**21**	High	None	S	3.98	0.85
**22**	High	None	None	4.97	0.83
**23**	High	None	None	5.13	0.79
**24**	High	Reproductive	None	5.23	0.49
**25**	High	Mutagenic	S	5.03	0.54
Benznidazole	High	None	I, S	−1.66	0.34
Amphotericin B	Low	None	I	−0.14	0.37
Colchicine	High	None	S	1.02	0.45

^a^ Human intestinal absorption. ^b^ Evaluated for mutagenic, tumorigenic, skin irritant, and reproductive risks. ^c^ Interaction with P-gp: S = substrate, I = inhibitor, upper case letter = high affinity, lower case letter = moderate affinity. ^d^ Drug likeness. ^e^ Drug score.

**Table 8:** Binding constant values for the interaction between HSA and compound **23** in PBS medium.

	Steady-State Fluorescence						Time-Resolved Fluorescence		
T (K)	*K_SV_* × 10^5^ (M^−1^)	*k_q_* × 10^13^ (M^−1^s^−1^)	*n*	Δ*H*° (kJmol^−1^)	Δ*S*° (kJmol^−1^K^−1^)	Δ*G*° (kJmol^−1^)	Parameters	HSA	HSA:23
289	1.56 ± 0.02	3.22	0.992 ± 0.01	−7.85 ± 0.71	72.2 ± 2.3	−28.7	τ_1_ (ns)	1.65 ± 0.10	1.59 ± 0.11
296	1.44 ± 0.02	2.97	1.04 ± 0.01			−29.2	%Relative τ_1_	22.0	23.5
303	1.31 ± 0.07	2.70	1.09 ± 0.01			−29.7	τ_2_ (ns)	5.75 ± 0.10	5.70 ± 0.12
310	1.22 ± 0.01	2.52	1.02 ± 0.01			−30.2	%Relative τ_2_	78.0	76.5
317	1.19 ± 0.02	2.45	1.02 ± 0.01			−30.7	τ_average_ (ns)	4.85 ± 0.10	4.73 ± 0.11

## Data Availability

All analyzed data are contained in the main text and in the Supplementary Material of the article. Raw data are available from the authors upon request.

## Supplementary Materials

The following supporting information can be downloaded at https://www.mdpi.com/article/10.3390/tropicalmed7120403/s1, Figures S1–S34: Copies of ^1^H and ^13^C NMR spectra for compounds **9**–**25**; Table S1: In vitro percent viability of mouse splenocytes after treatment with compounds **9**–**25** at 100 μM compared with the untreated control. Table S2: In vitro dose-dependent viability of mouse splenocytes after treatment with compounds **15** and **23**.
