# Supplementary material for: A Novel Protocol for the Synthesis of 1,2,4-Oxadiazoles Active against Trypanosomatids and Drug-Resistant Leukemia Cell Lines
Source: Trop Med Infect Dis. 2022 Nov 28;7(12):403. doi: 10.3390/tropicalmed7120403 (PMC9787607; doi:10.3390/tropicalmed7120403)

Supplemental Material

1. Copies of ^{1}H and DEPTQ ^{13}C NMR spectra for compounds 9–25

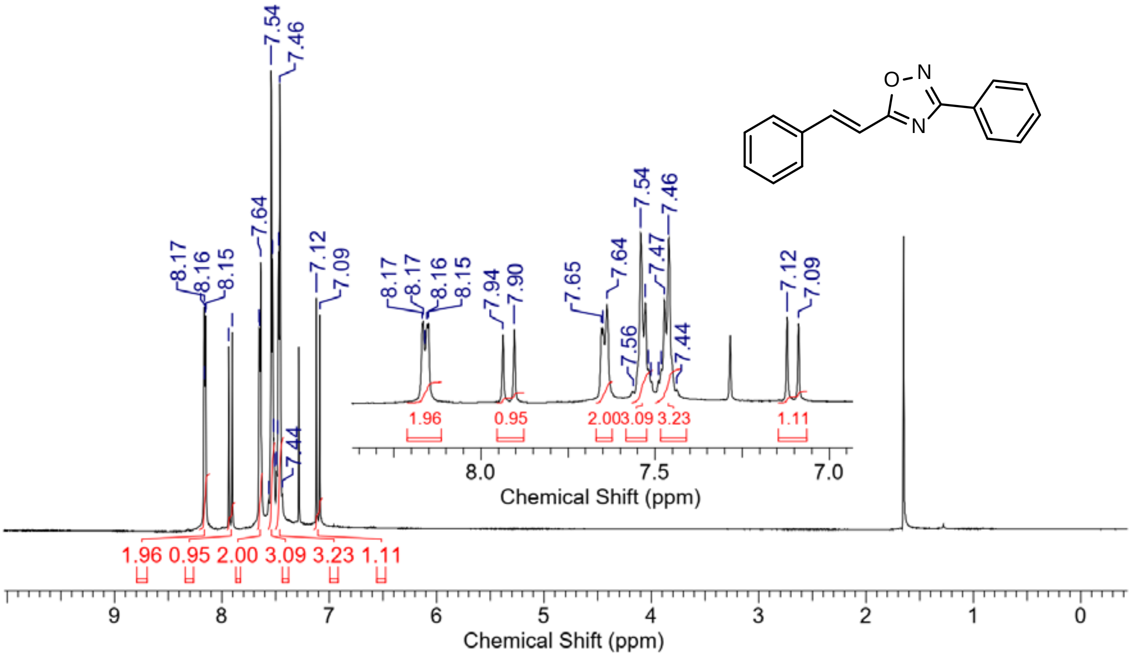

Figure S1. – ^{1}H NMR spectrum of compound **9.**

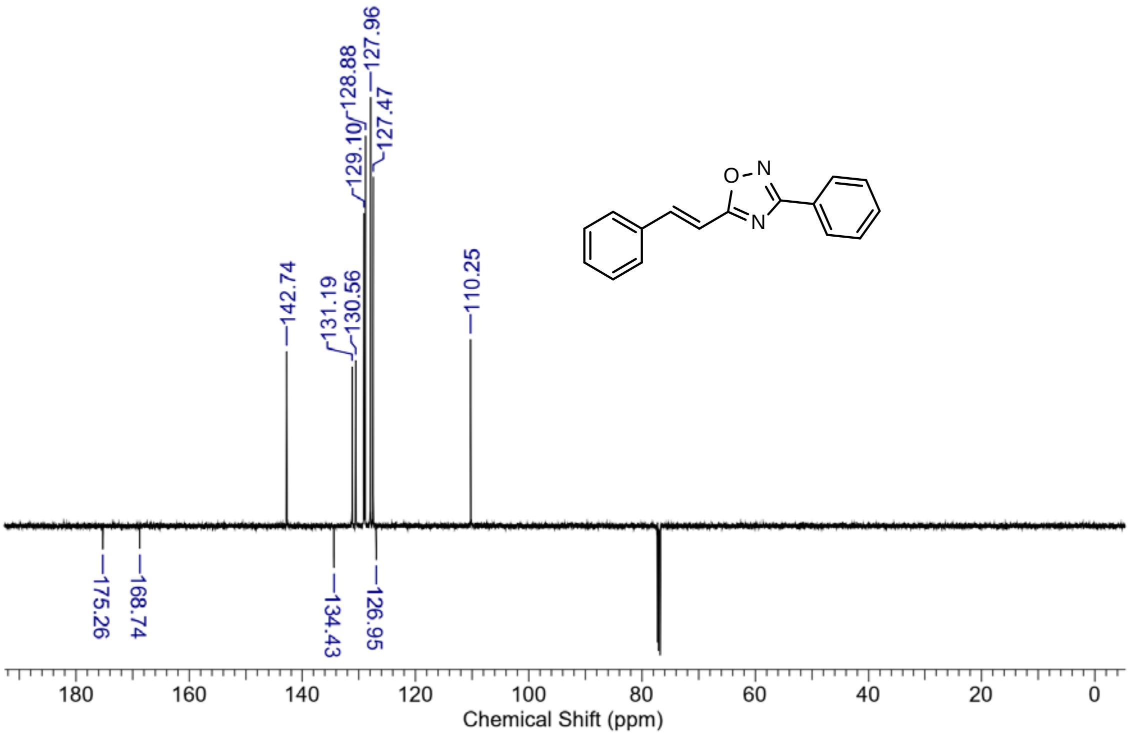

Figure S2. – DEPTQ ^{13}C NMR spectrum of compound **9.**

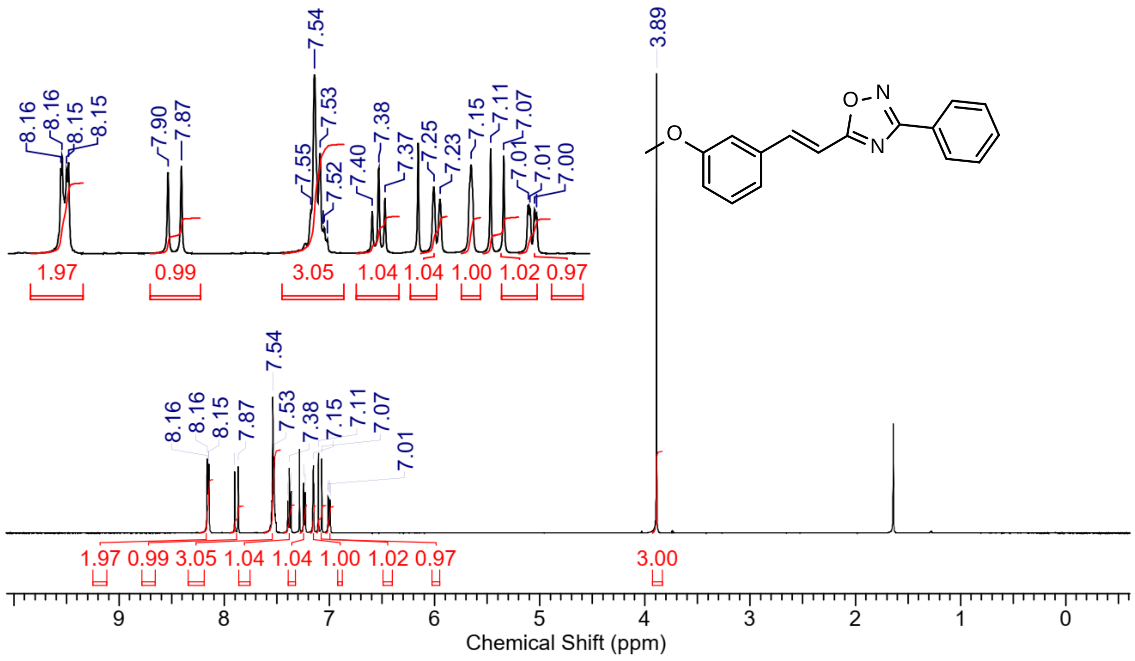

Figure S3. – ^{1}H NMR spectrum of compound **10.**

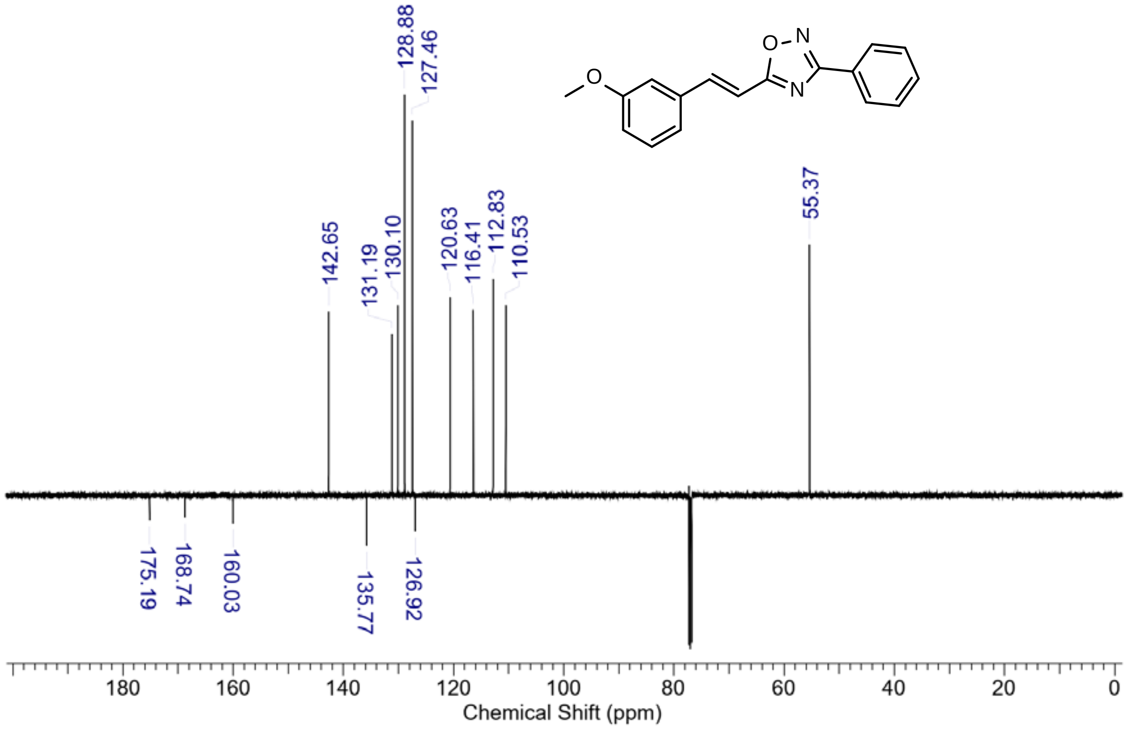

Figure S4. – DEPTQ ^{13}C NMR spectrum of compound **10.**

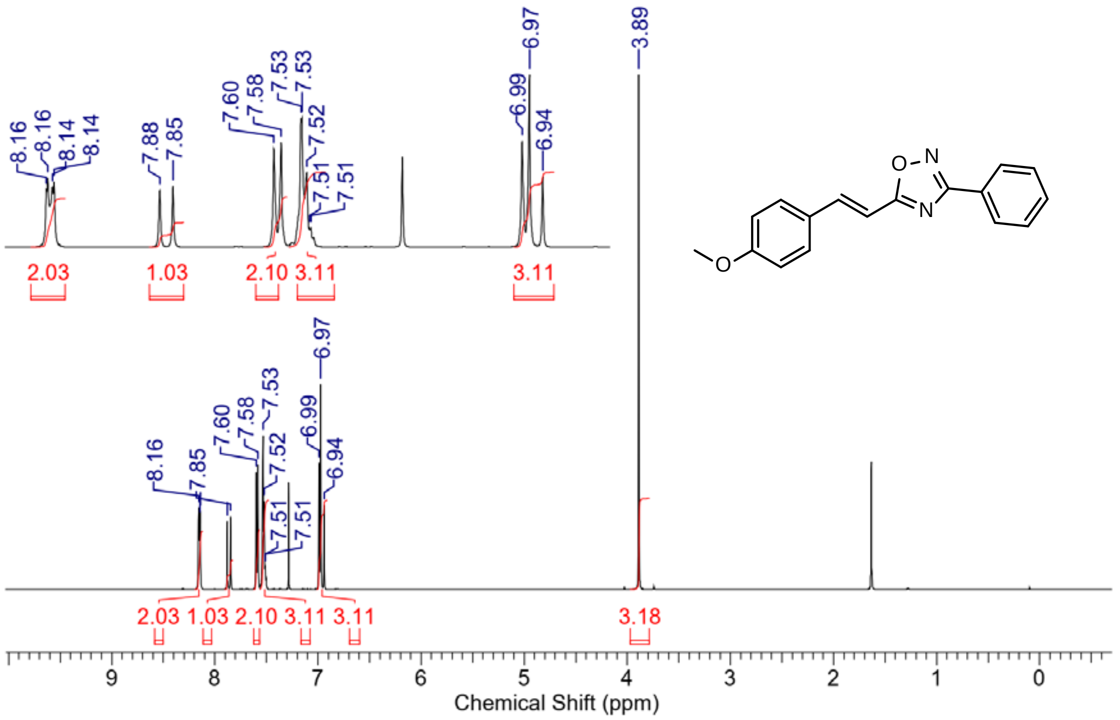

Figure S5. – ^{1}H NMR spectrum of compound **11.**

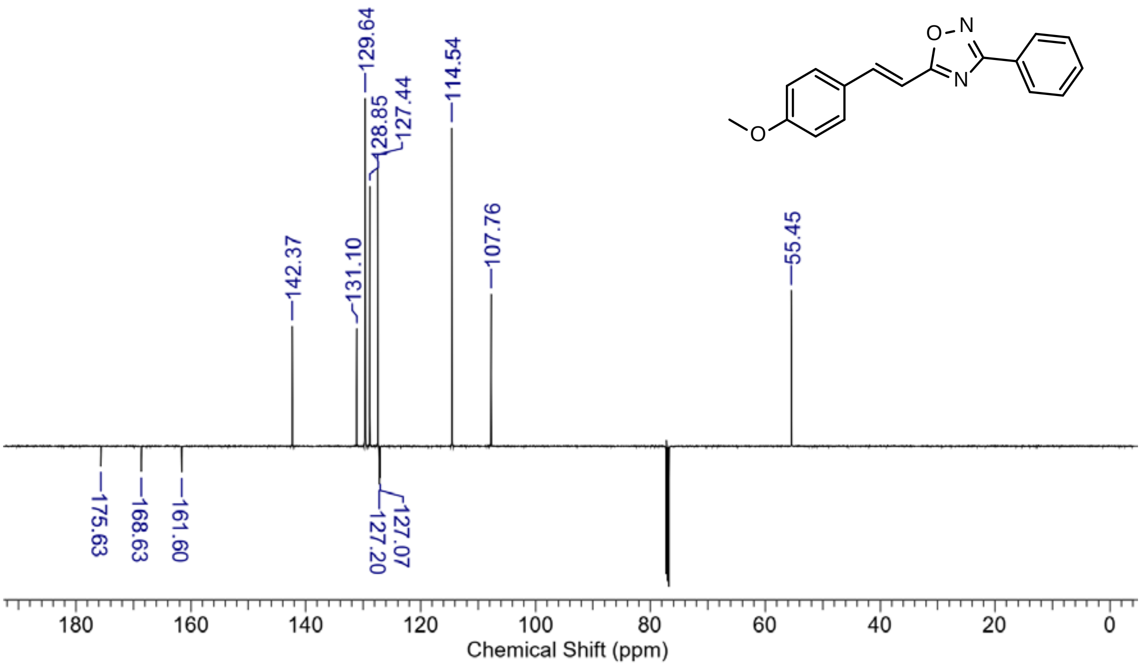

Figure S6. – DEPTQ ^{13}C NMR spectrum of compound **11.**

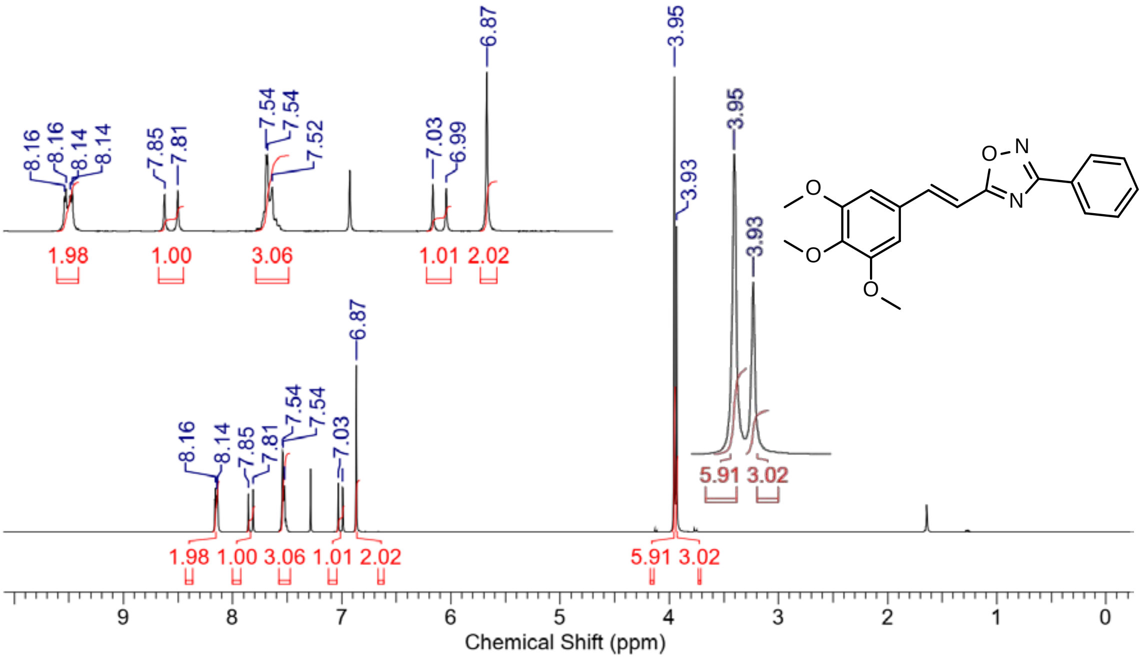

Figure S7. – ^{1}H NMR spectrum of compound **12.**

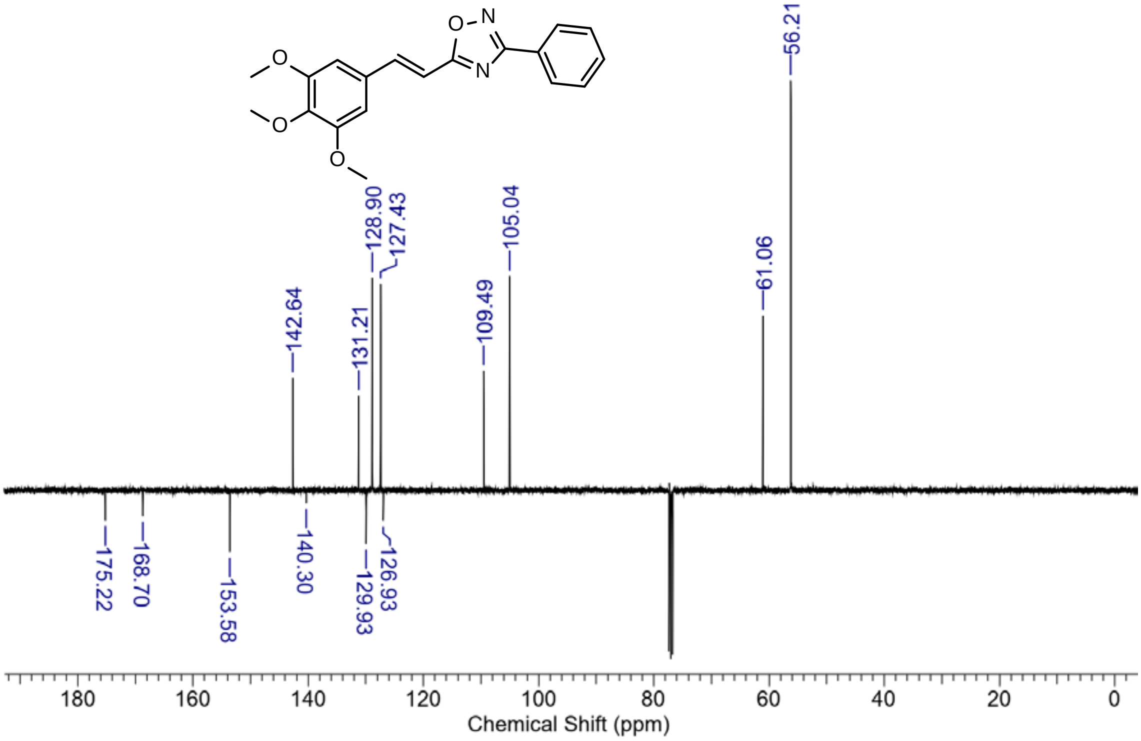

Figure S8. – DEPTQ ^{13}C NMR spectrum of compound **12.**

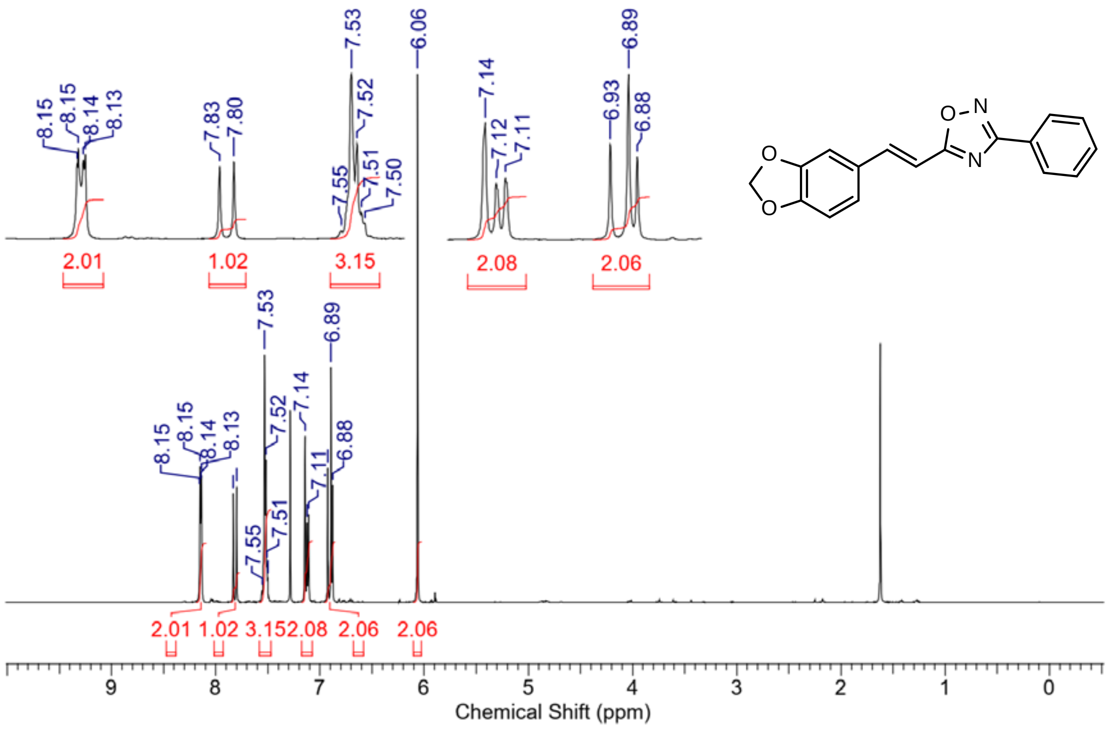

Figure S9. – ^{1}H NMR spectrum of compound **13.**

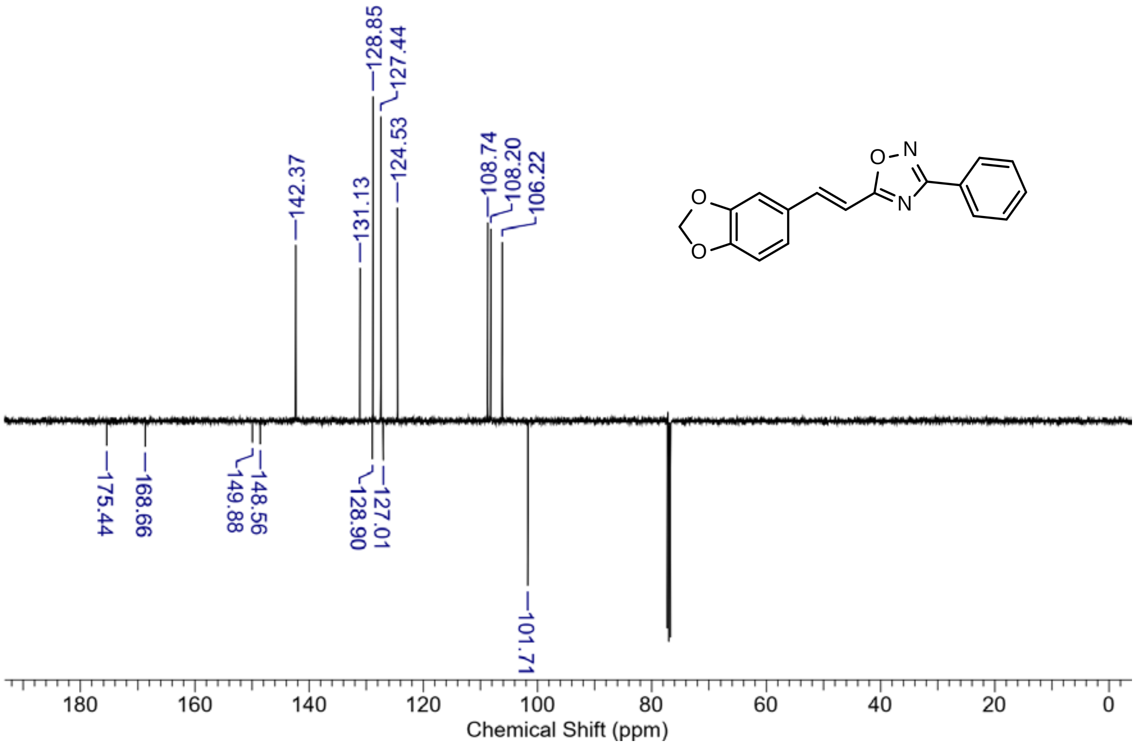

Figure S10. – DEPTQ ^{13}C NMR spectrum of compound **13.**

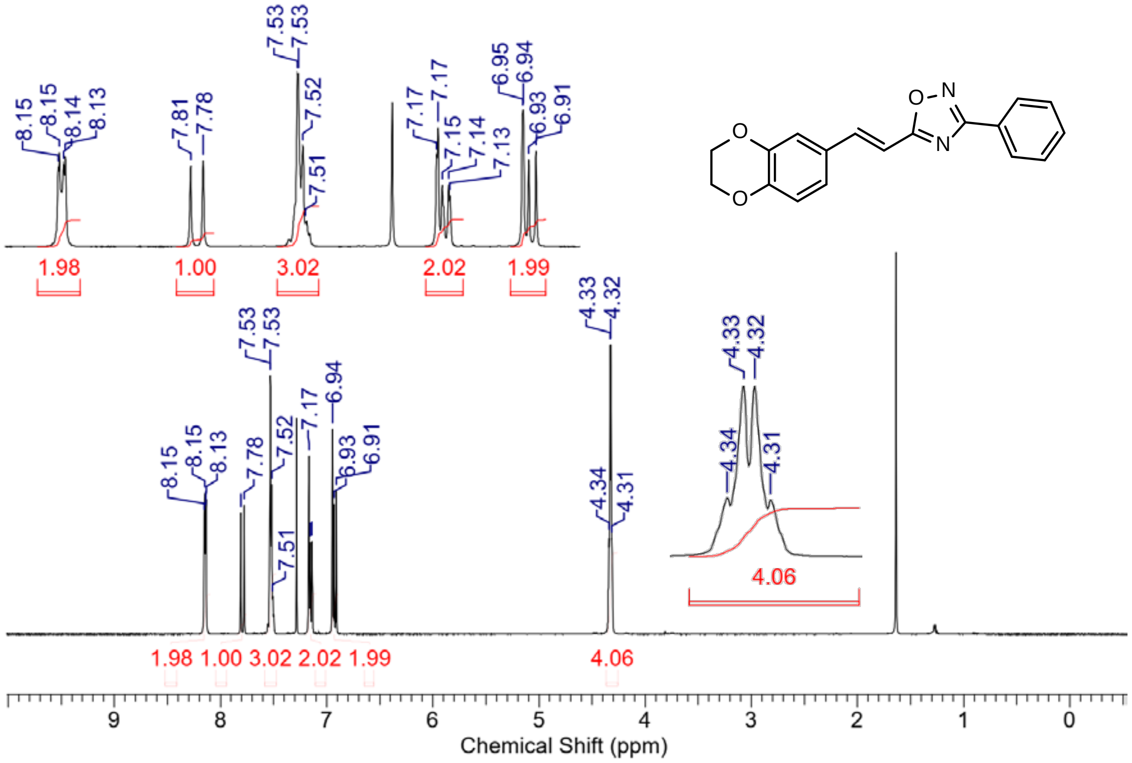

Figure S11. – ^{1}H NMR spectrum of compound **14.**

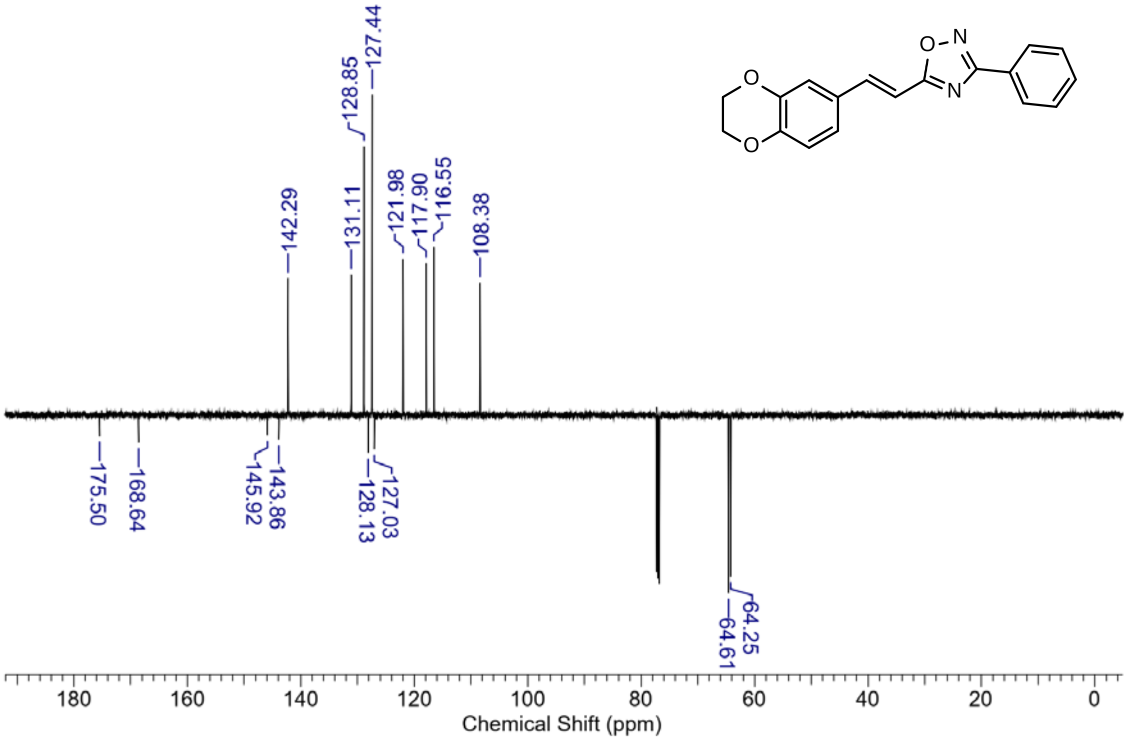

Figure S12. – DEPTQ ^{13}C NMR spectrum of compound **14.**

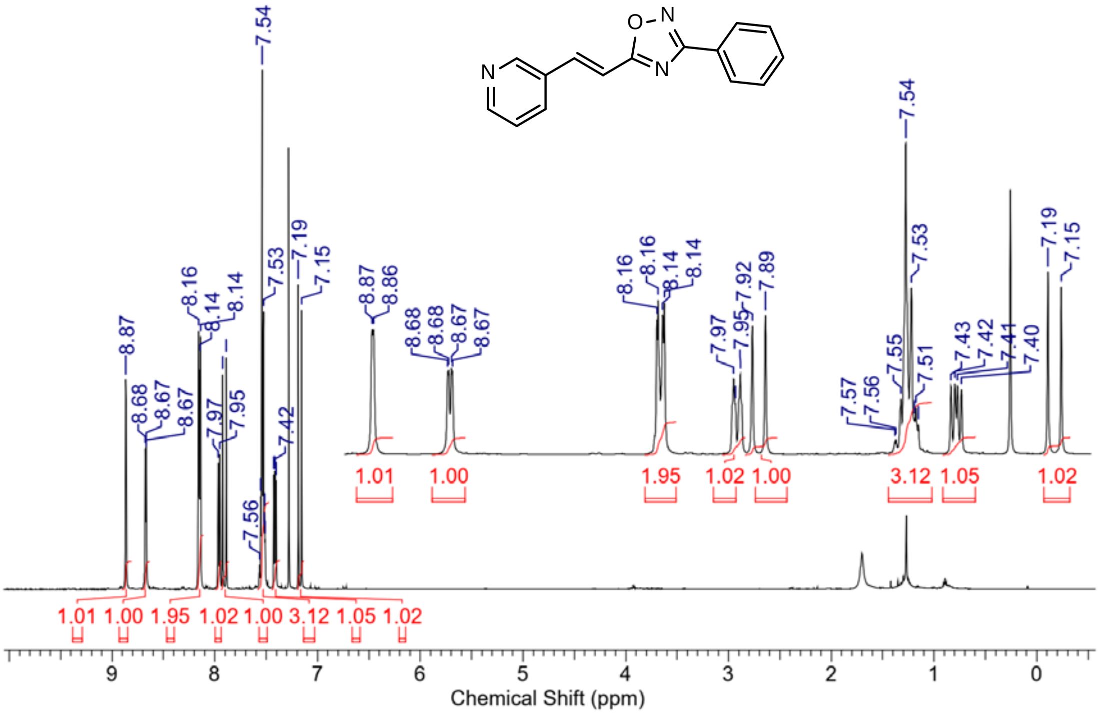

Figure S13. – ^{1}H NMR spectrum of compound **15.**

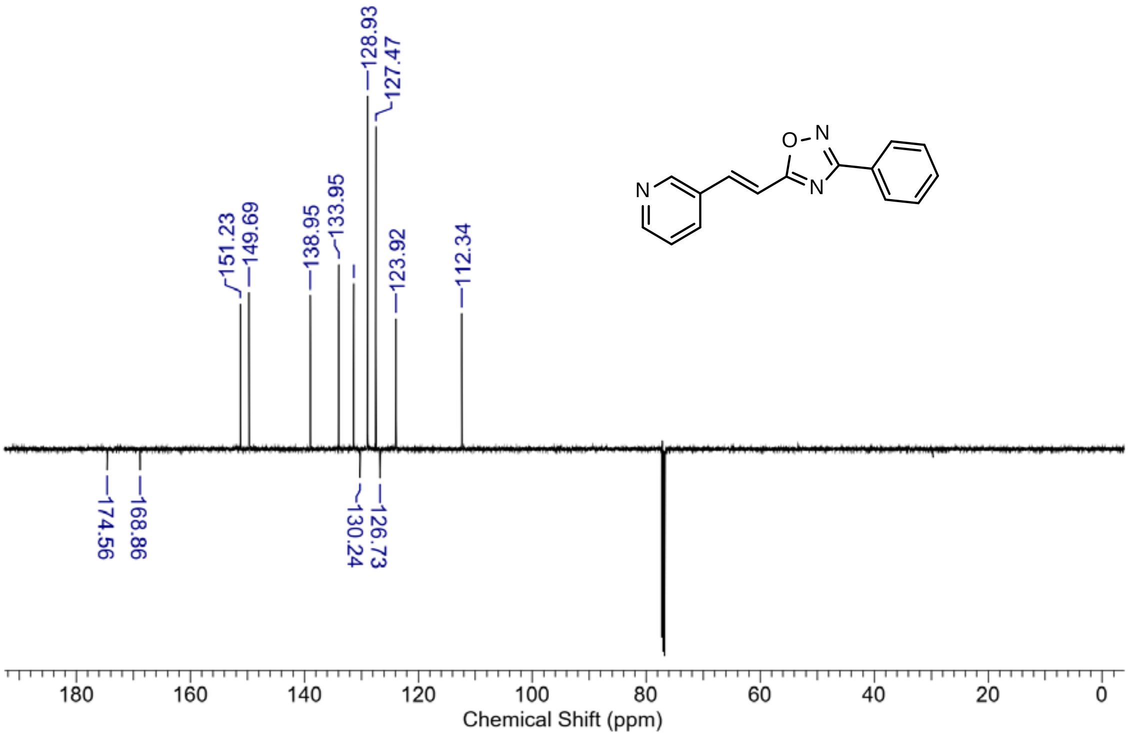

Figure S14. – DEPTQ ^{13}C NMR spectrum of compound **15.**

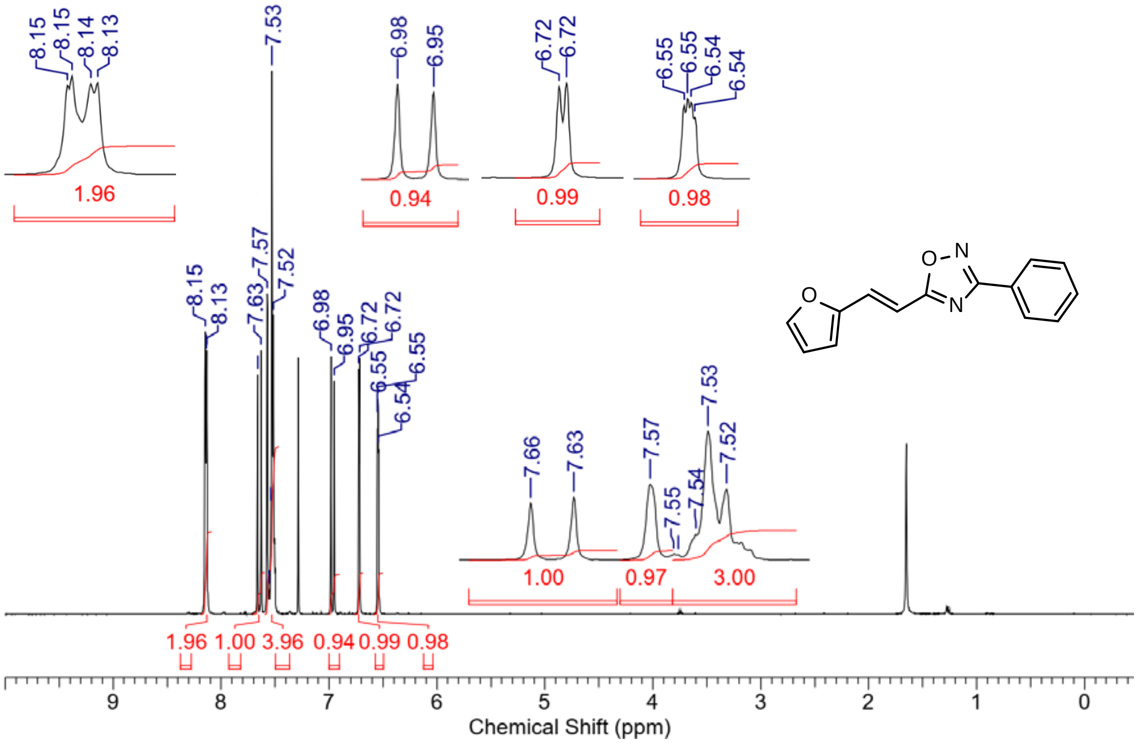

Figure S15. – ^{1}H NMR spectrum of compound **16.**

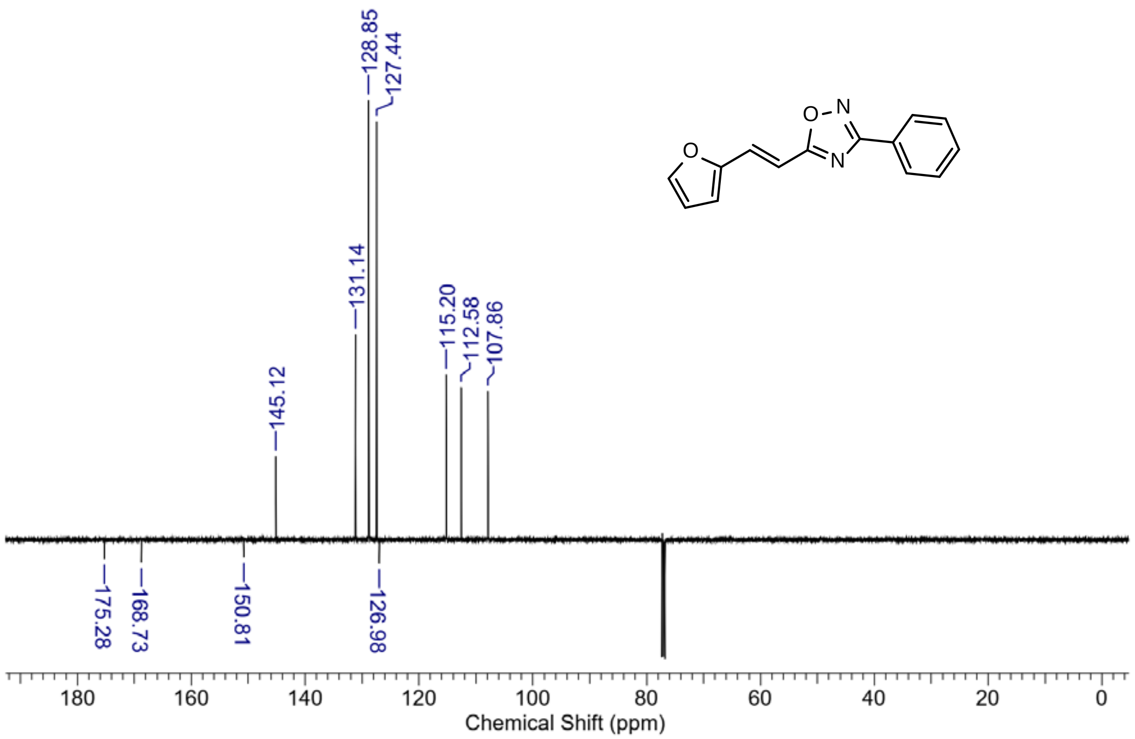

Figure S16. – DEPTQ ^{13}C NMR spectrum of compound **16.**

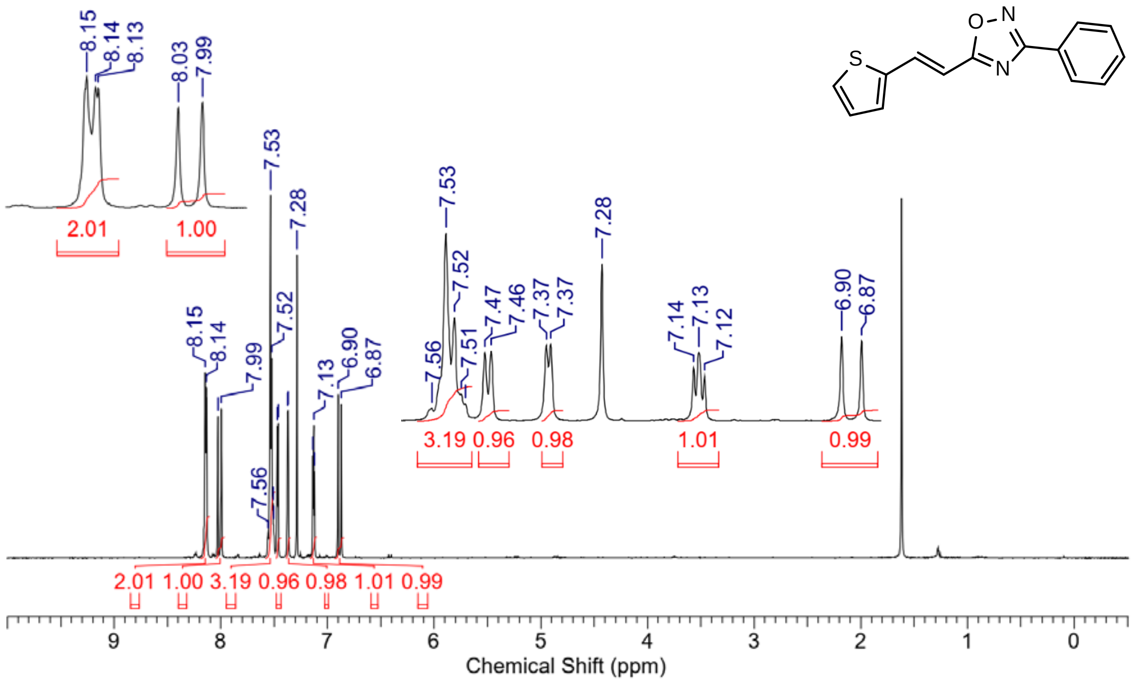

Figure S17. – ^{1}H NMR spectrum of compound **17.**

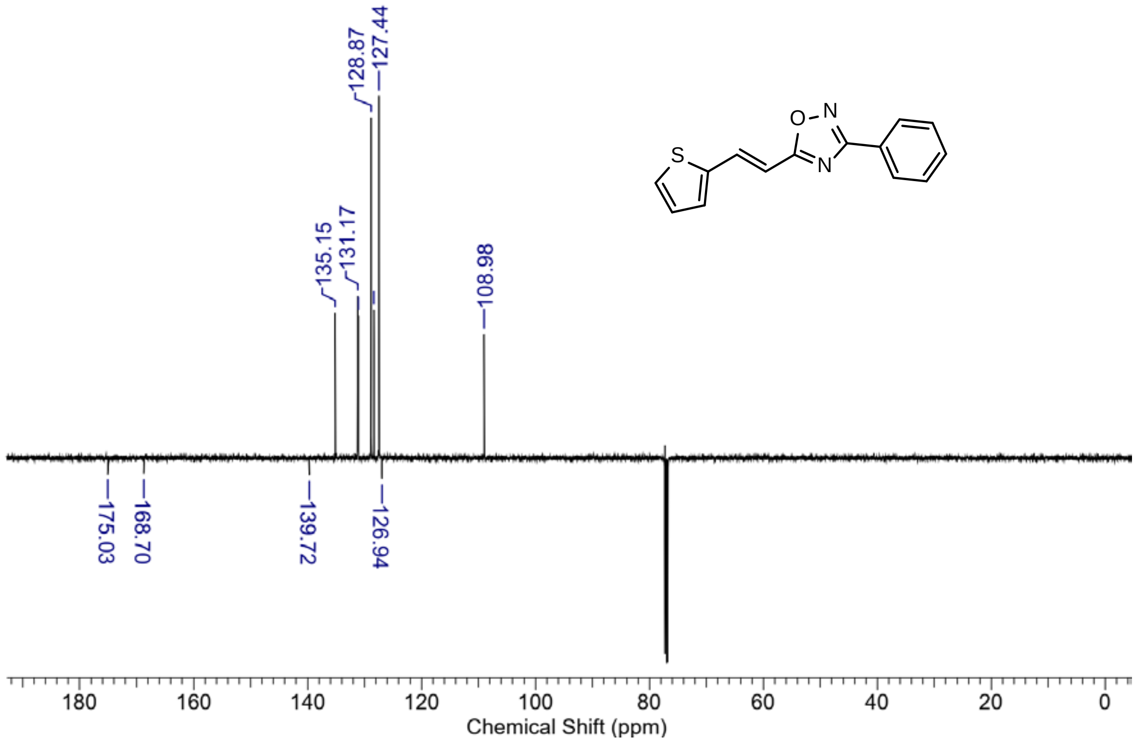

Figure S18. – DEPTQ ^{13}C NMR spectrum of compound **17.**

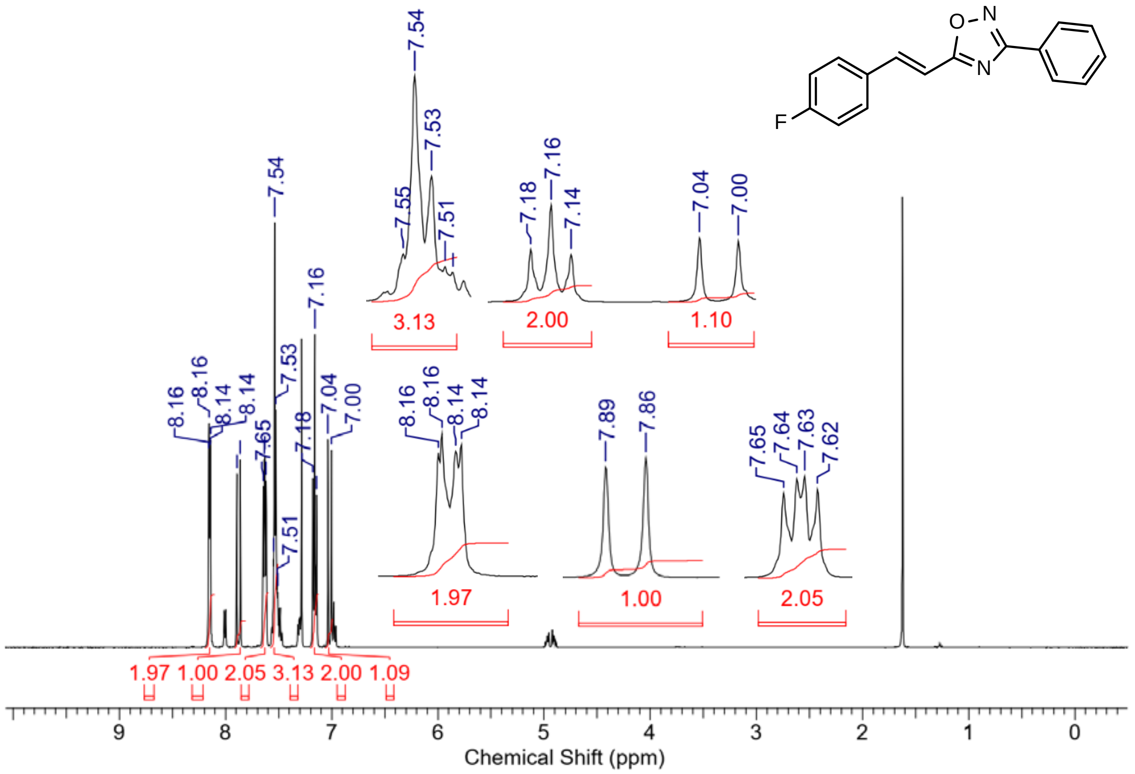

Figure S19. – ^{1}H NMR spectrum of compound **18.**

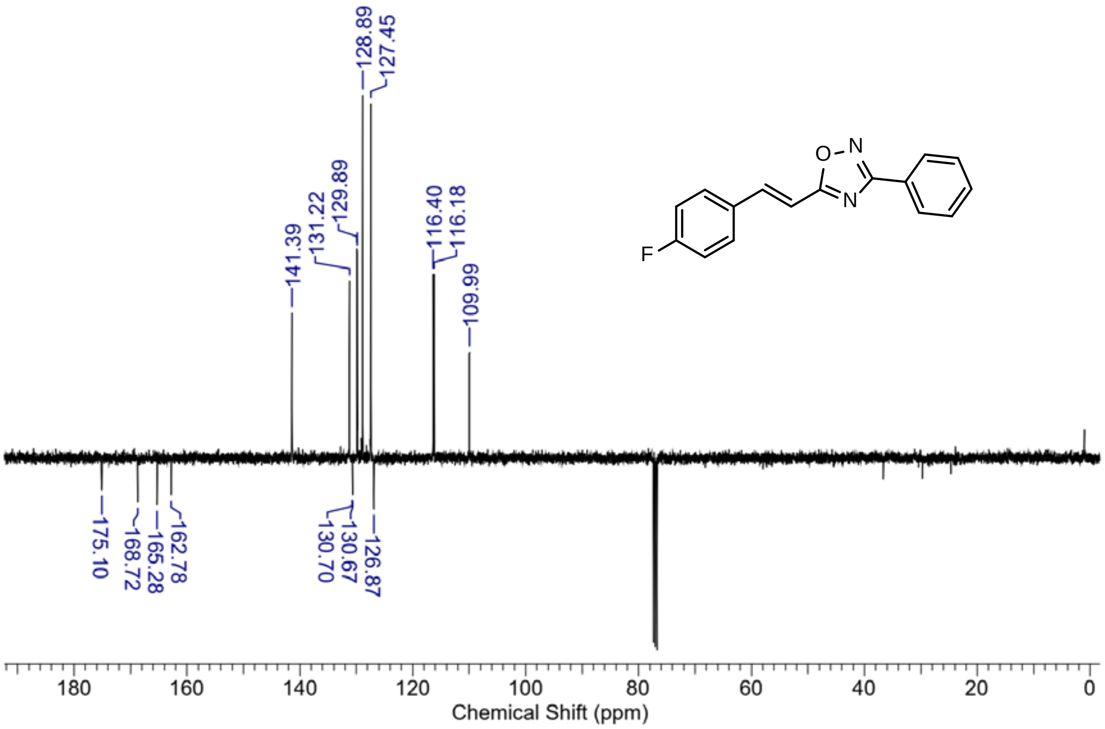

Figure S20. – DEPTQ ^{13}C NMR spectrum of compound **18** .

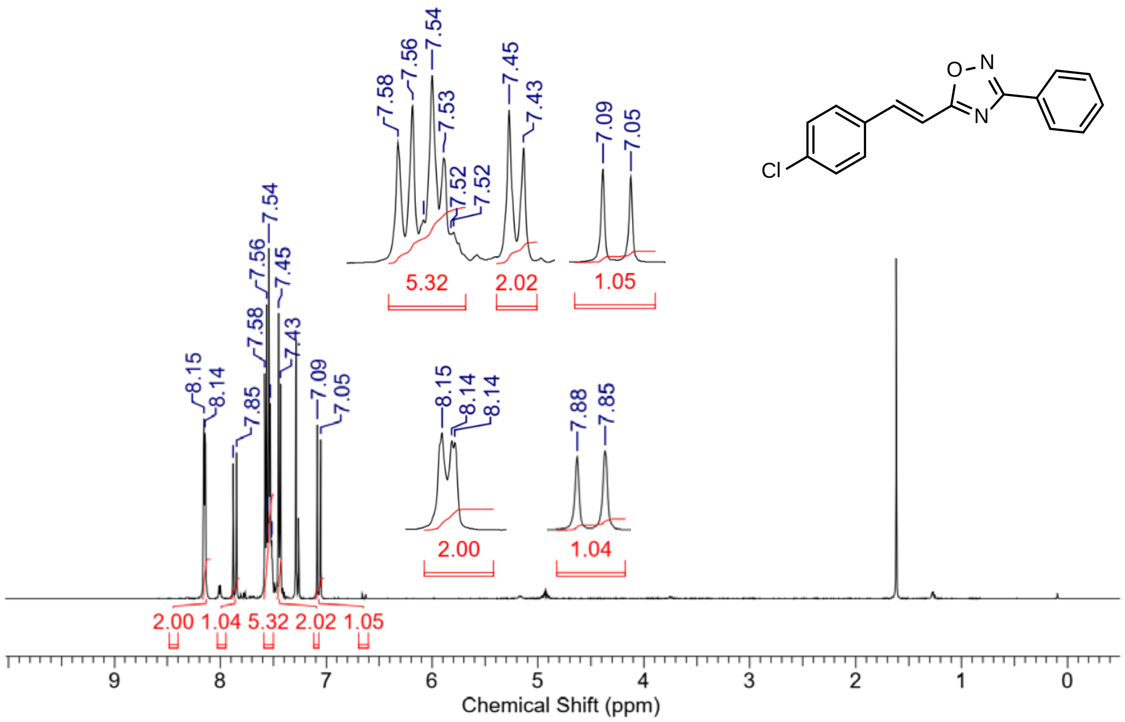

Figure S21. – ^{1}H NMR spectrum of compound **19.**

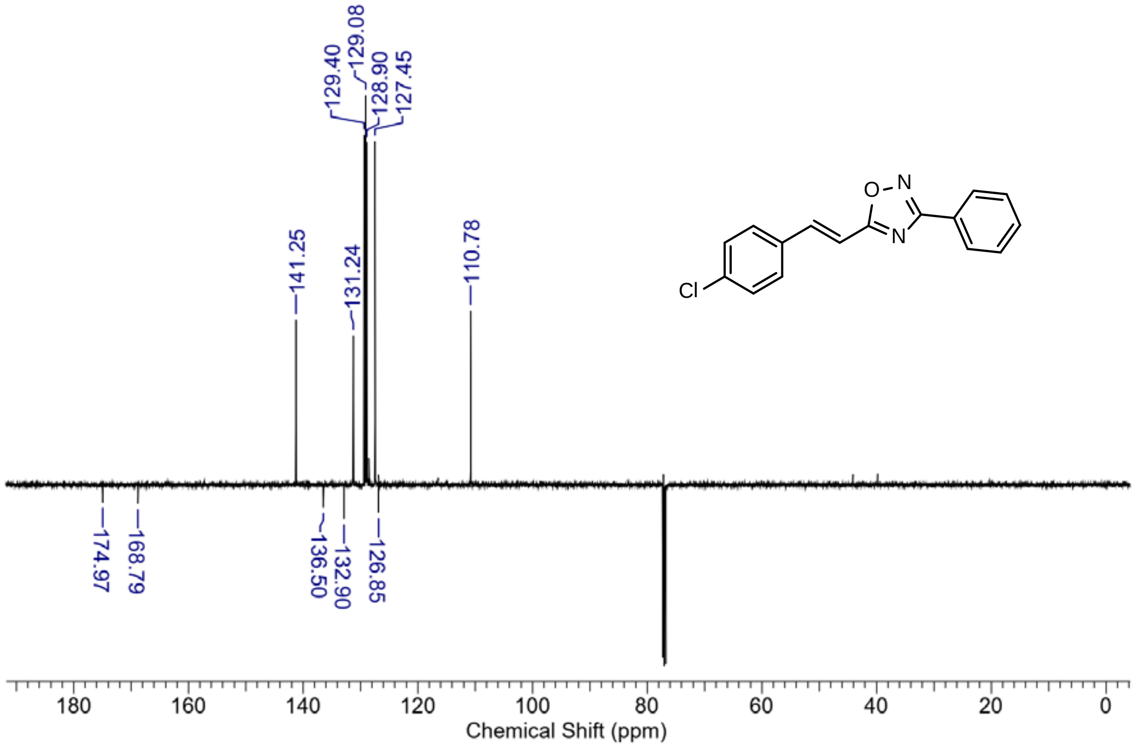

Figure S22. – DEPTQ ^{13}C NMR spectrum of compound **19.**

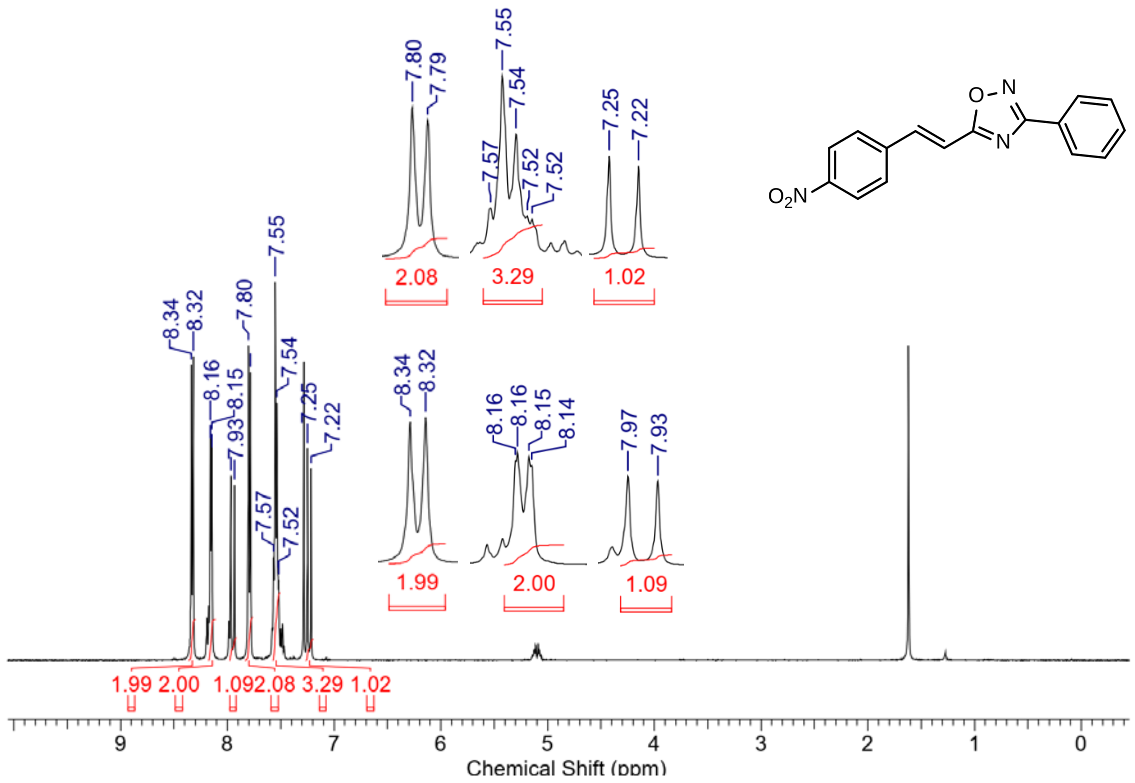

Figure S23. – ^{1}H NMR spectrum of compound **20.**

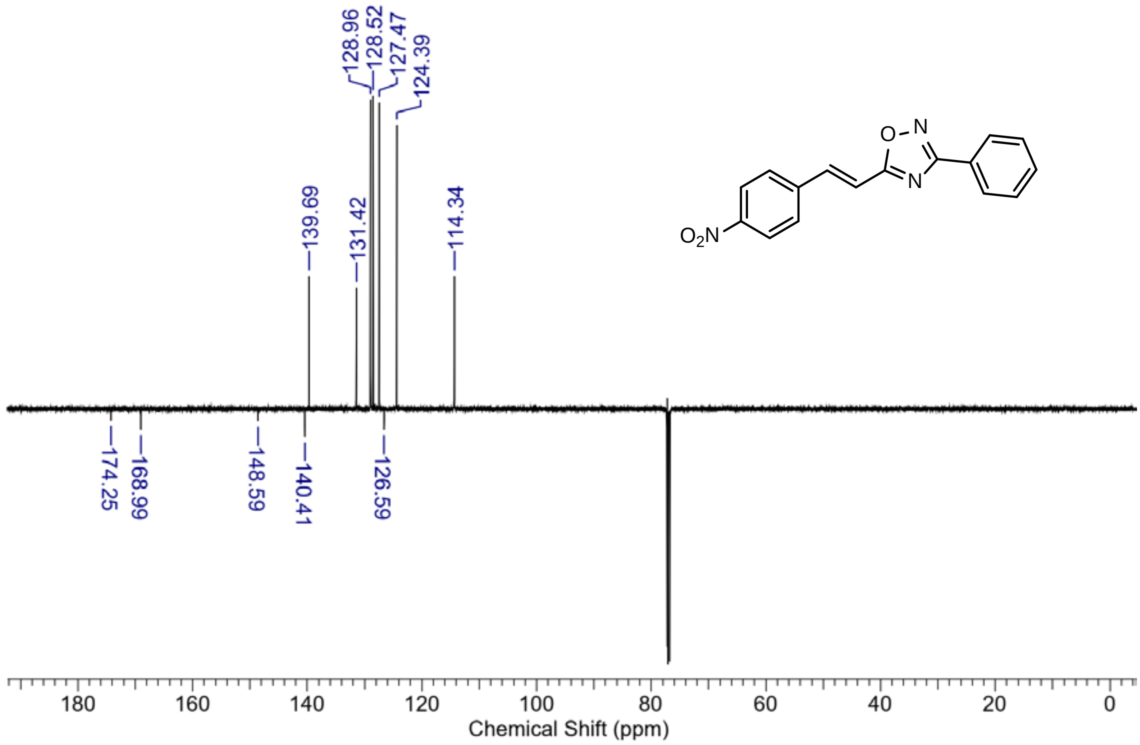

Figure S24. – DEPTQ ^{13}C NMR spectrum of compound **20.**

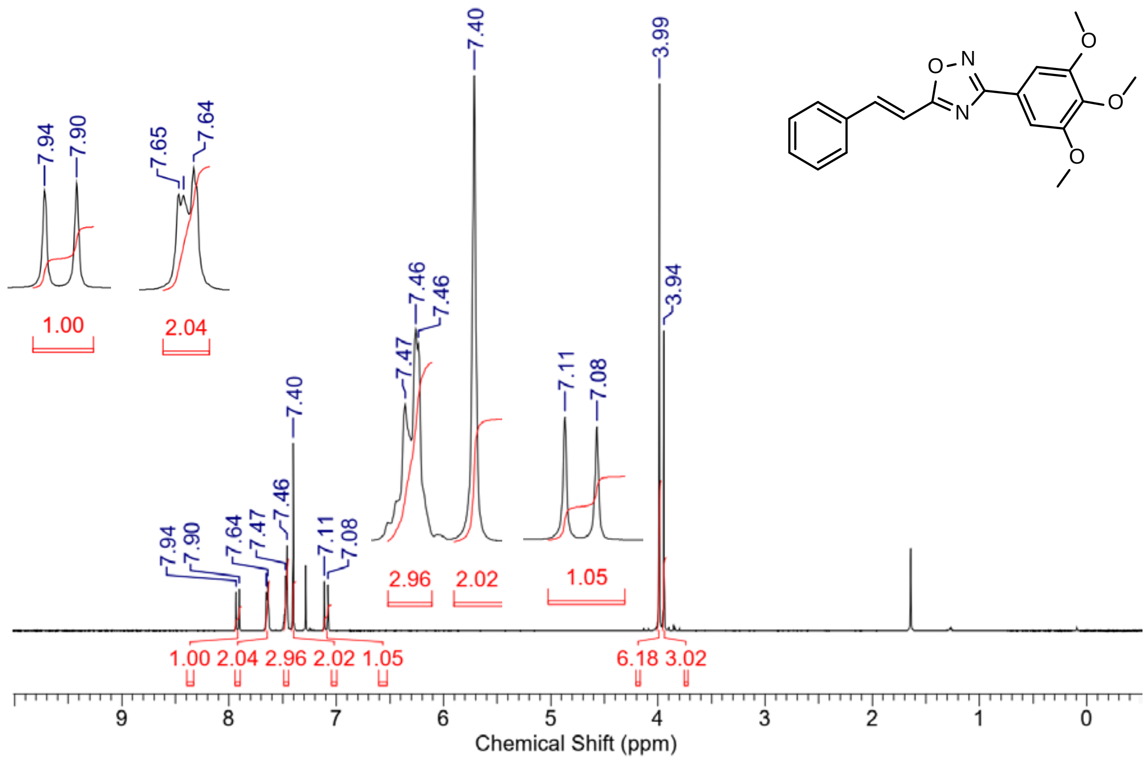

Figure S25. – ^{1}H NMR spectrum of compound **21.**

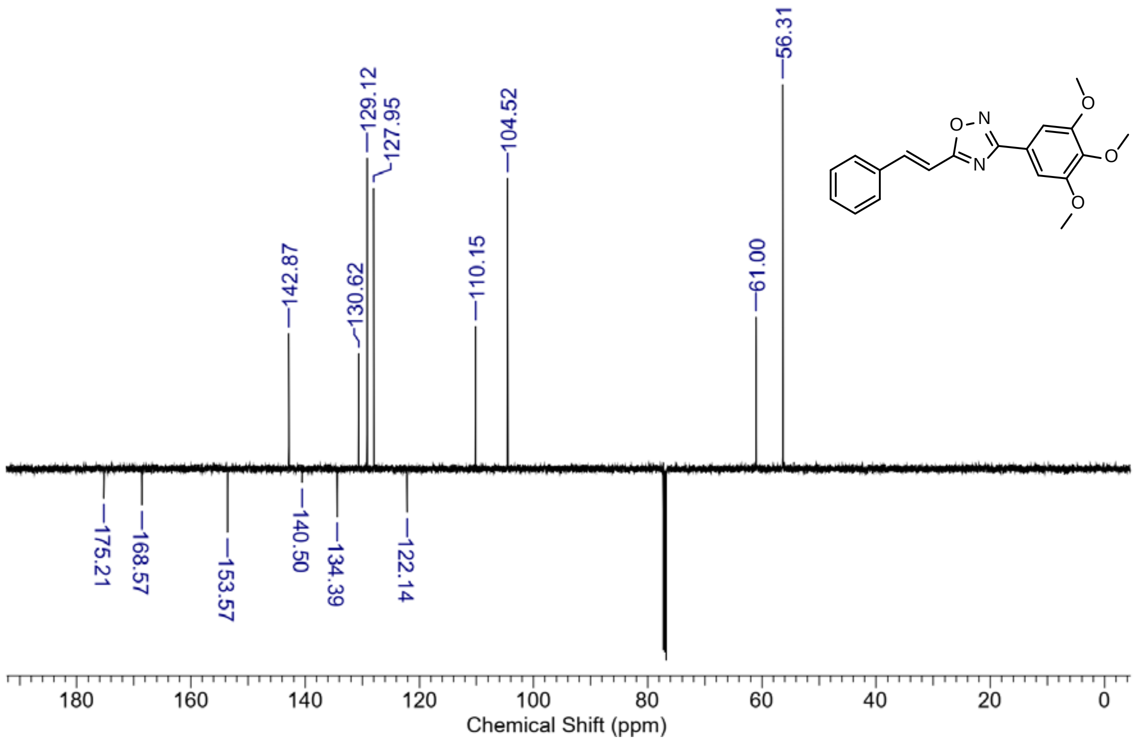

Figure S26. – DEPTQ ^{13}C NMR spectrum of compound **21.**

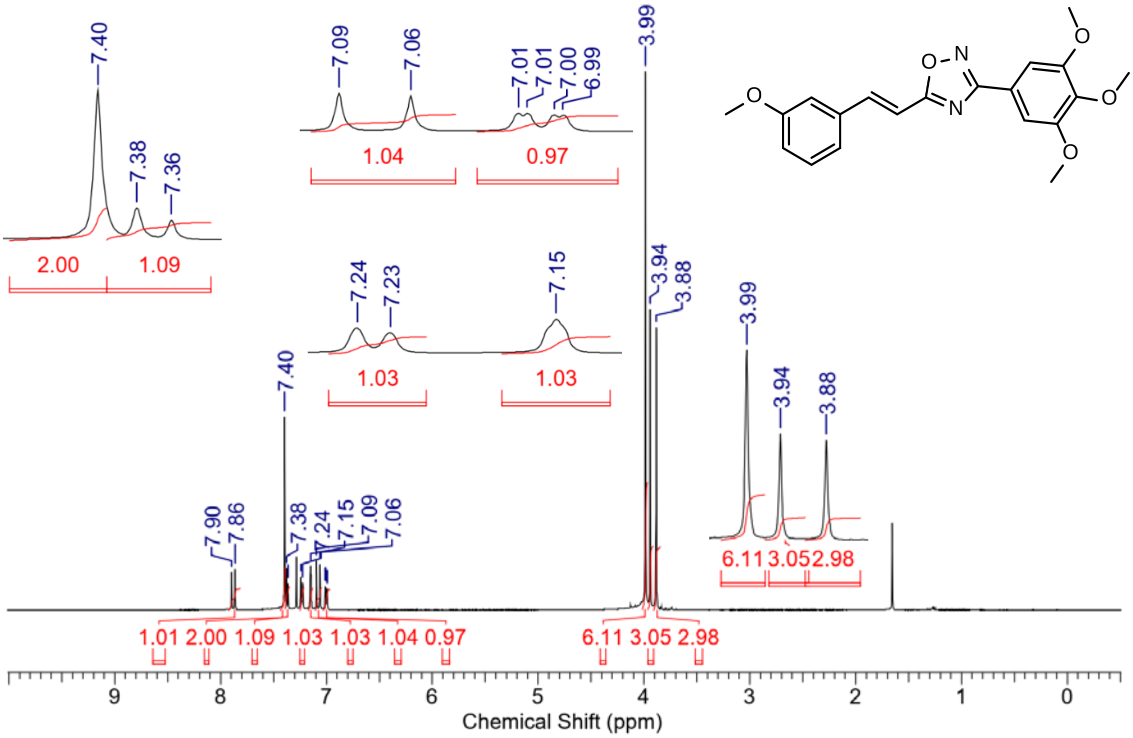

Figure S27. – ^{1}H NMR spectrum of compound **22.**

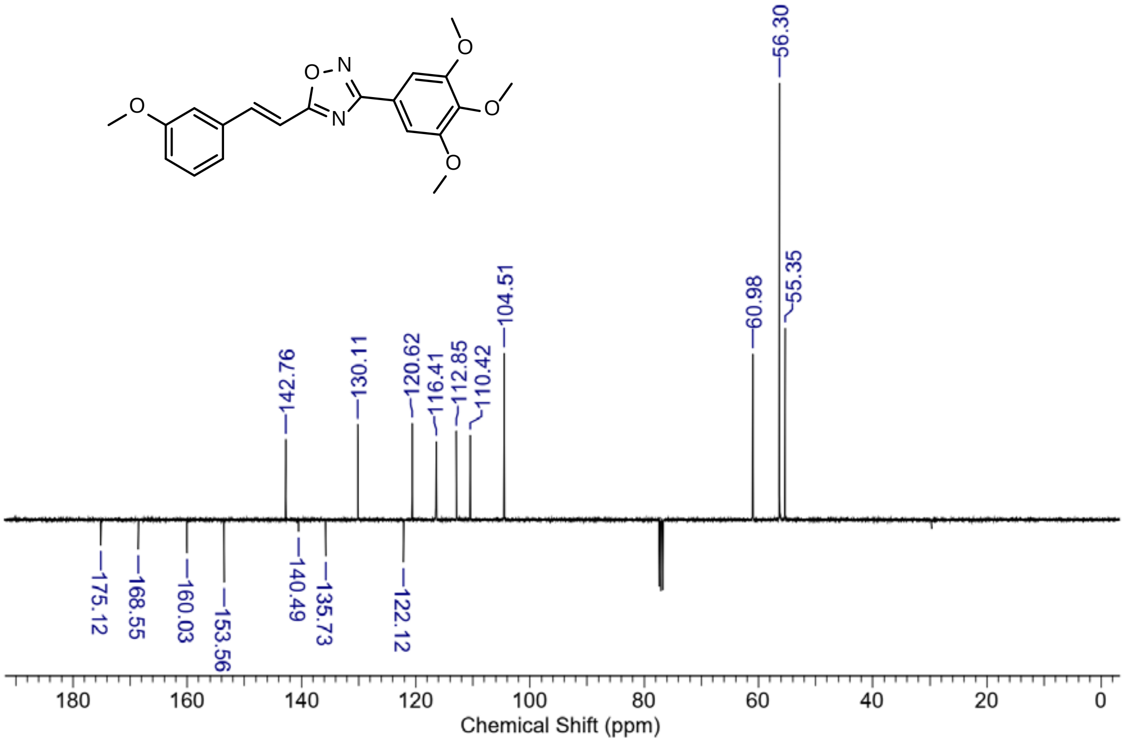

Figure S28. – DEPTQ ^{13}C NMR spectrum of compound **22.**

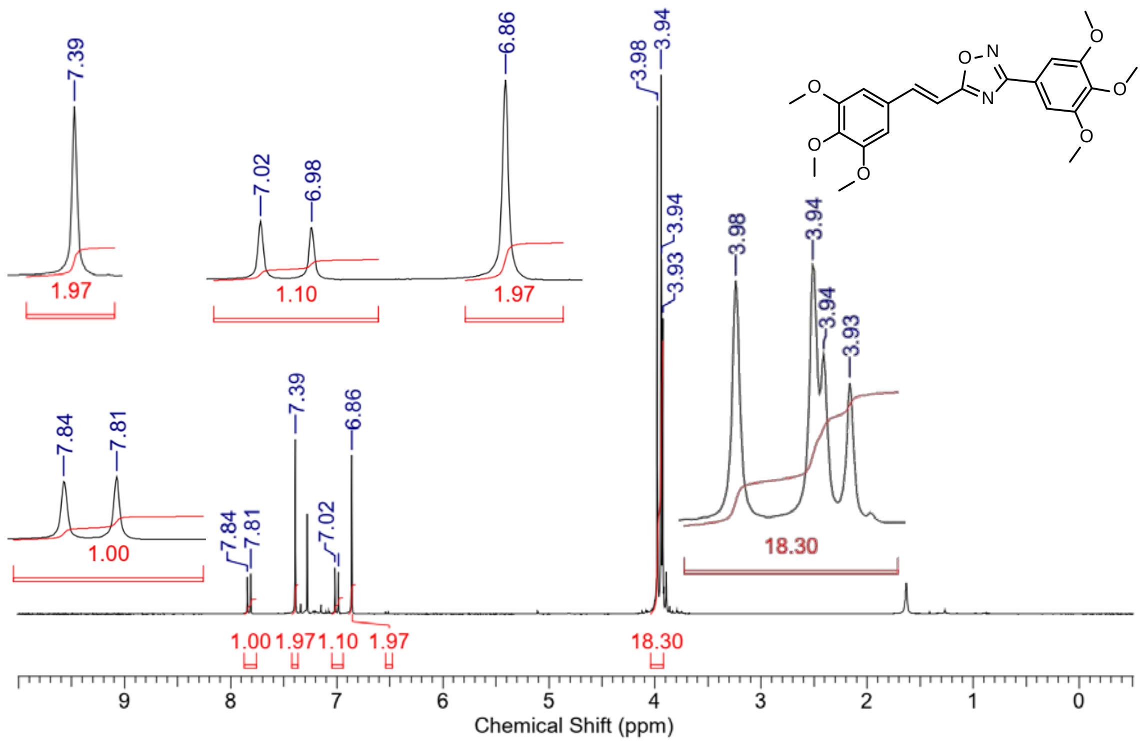

Figure S29. – ^{1}H NMR spectrum of compound **23.**

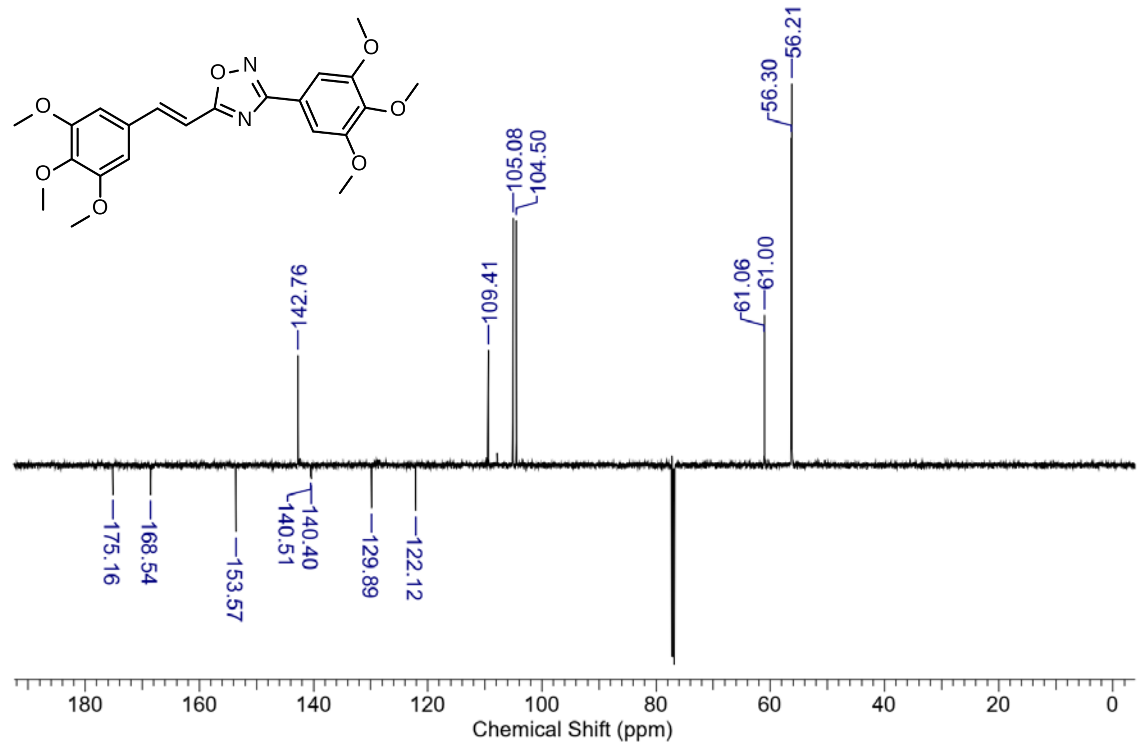

Figure S30. – DEPTQ ^{13}C NMR spectrum of compound **23.**

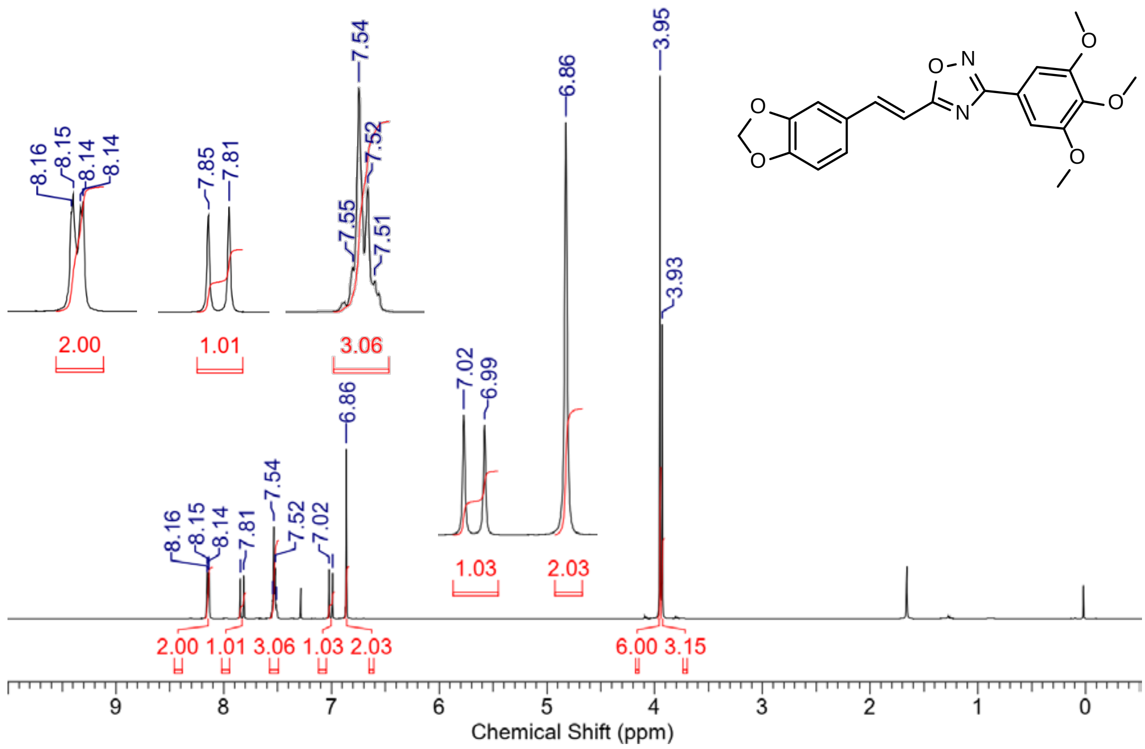

Figure S31. – ^{1}H NMR spectrum of compound **24.**

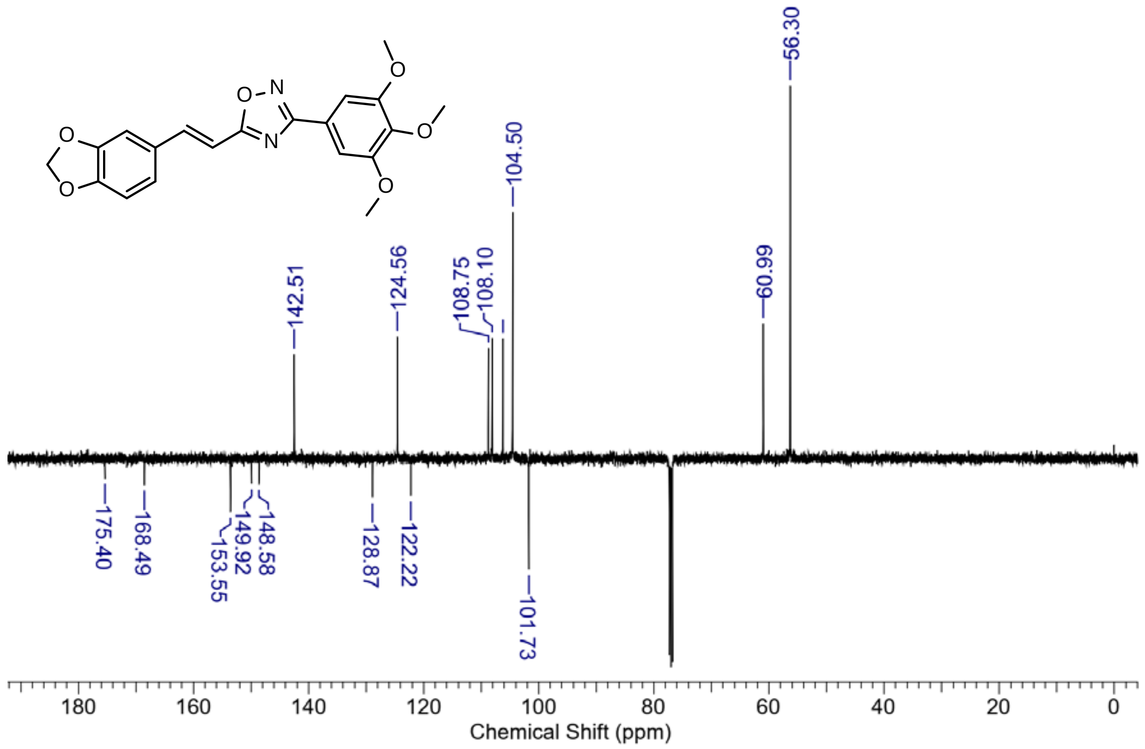

Figure S32. – DEPTQ ^{13}C NMR spectrum of compound **24.**

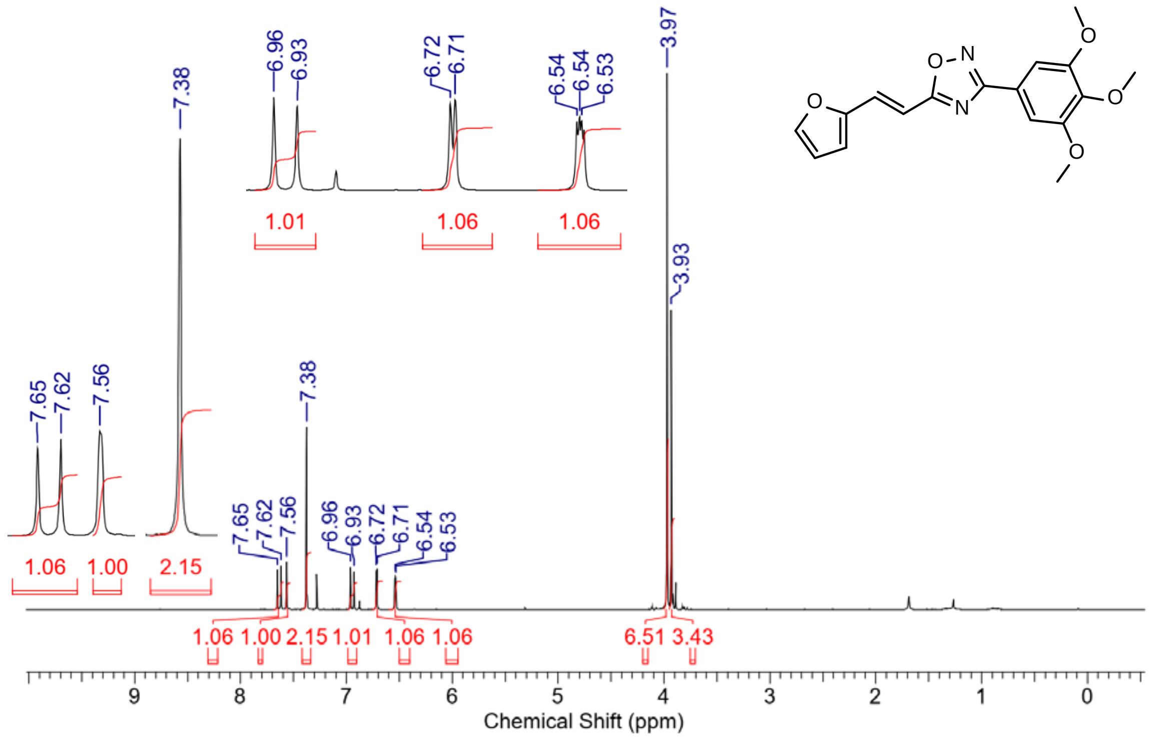

Figure S33. – ^{1}H NMR spectrum of compound **25.**

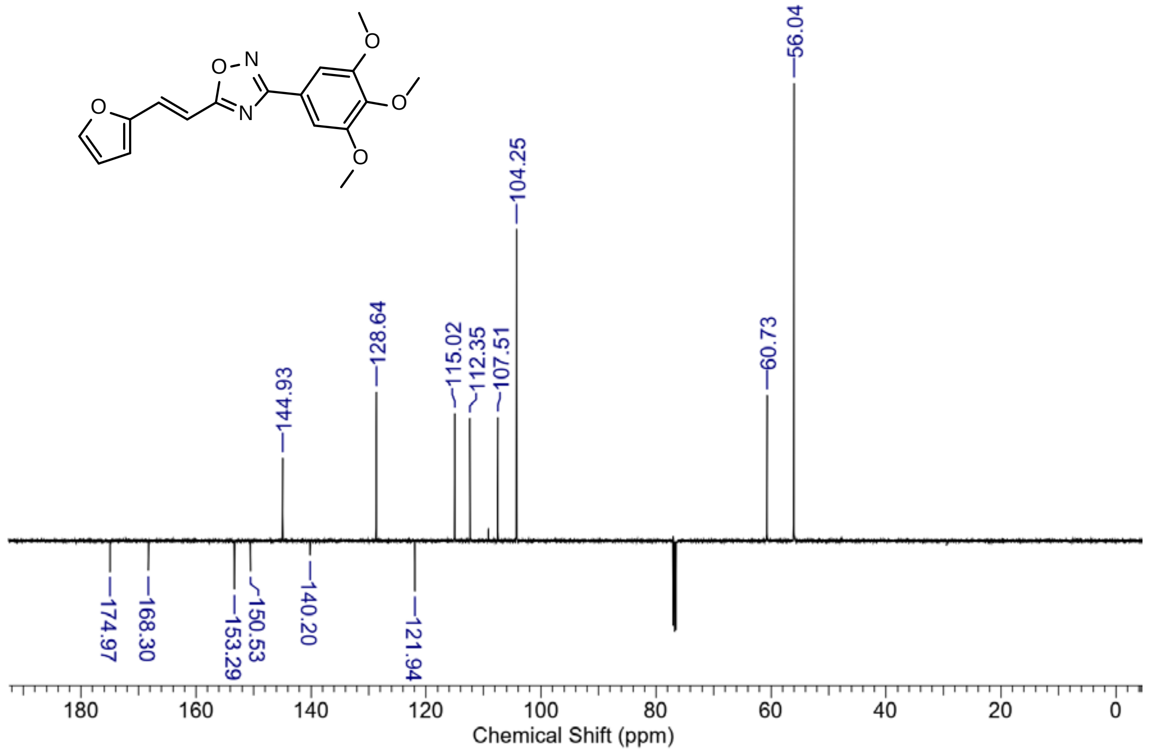

Figure S34. – DEPTQ ^{13}C NMR spectrum of compound **25.**

Table S1. *In vitro* percent viability of mouse splenocytes after treatment with compounds **15 – 31** at 100 µM compared to the untreated control.

Compound	% Viability[a]	Compound	% Viability
**9**	102.1 ± 7.4	**18**	101.5 ± 11.5
**10**	85.4 ± 4.0	**19**	90.5 ± 5.3
**11**	84.4 ± 2.9	**20**	77.8 ± 4.2
**12**	72.1 ± 6.5	**21**	76.8 ± 6.6
**13**	98.8 ± 5.1	**22**	63.5 ± 2.4
**14**	93.6 ± 5.9	**23**	14.1 ± 3.5
**15**	29.5 ± 1.6	**24**	72.8 ± 4.2
**16**	53.5 ± 5.2	**25**	63.7 ± 4.7
**17**	99.0 ± 8.4		

[a] Compared to the untreated control

Table S2. *In vitro* dose-dependent viability of mouse splenocytes after treatment with compounds **15** and **23**.

Conc. (µM)	% Viability
100	29.5 ± 1.6
40	63.1 ± 5.8
16	77.7 ± 4.8
6.4	90.2 ± 10.7
2.6	86.3 ± 7.5
1.0	92.7 ± 4.1
0.4	94.5 ± 9.1

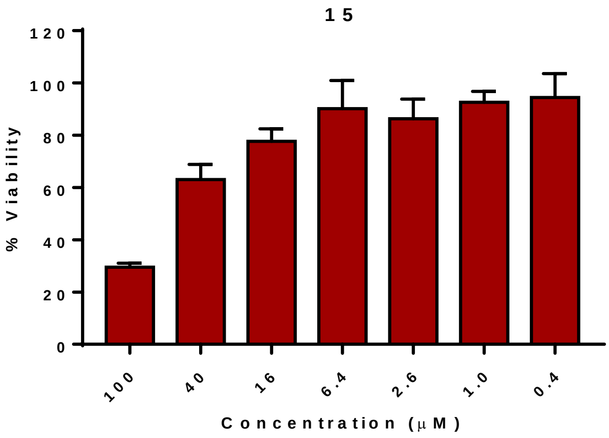

Conc. (µM)	% Viability
100	14.1 ± 3.5
40	43.4 ± 4.2
16	69.5 ± 6.4
6.4	84.6 ± 6.7
2.6	95.0 ± 4.4
1.0	94.9 ± 4.0
0.4	97.3 ± 3.3

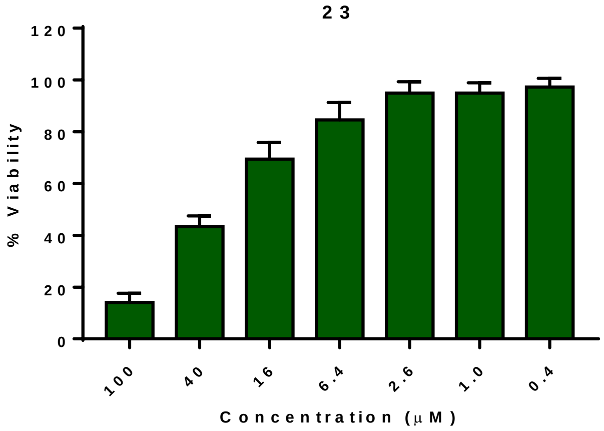

Supplement: Supplementary file 1 [file tropicalmed-07-00403-s001.zip › tropicalmed-1984941-supplementary.pdf]
